# ZDHHC5-mediated S-palmitoylation of FAK promotes its membrane localization and epithelial-mesenchymal transition in glioma

**DOI:** 10.1186/s12964-023-01366-z

**Published:** 2024-01-17

**Authors:** Yang Wang, Na Shen, Yang Yang, Yuan Xia, Wenhao Zhang, Yu Lu, Zhicheng Wang, Ze Yang, Zhangjie Wang

**Affiliations:** 1 Center for Clinical Medical Research, the Affiliated Hospital of Nantong University, Nantong, 226001 China; 2 Department of Hematology, the First Affiliated Hospital of Nanjing Medical University, Nanjing, 210029 China; 3 Department of Pediatric Surgery, the Affiliated Hospital of Nantong University, Nantong, 226001 China; 4 Department of Thoracic Surgery, The First Affiliated Hospital of Nanjing Medical University, Nanjing, 210029 China; 5 Department of Orthopedics, the First Affiliated Hospital of Bengbu Medical College, Bengbu, 233099 China

**Keywords:** FAK ZDHHC5 S, Palmitoylation glioma epithelial, Mesenchymal transition

## Abstract

**Background:**

Abnormal activation of FAK is associated with tumor development and metastasis. Through interactions with other intracellular signalling molecules, FAK influences cytoskeletal remodelling, modulation of adhesion signalling, and activation of transcription factors, promoting migration and invasion of tumor cells. However, the exact mechanism that regulates these processes remains unresolved. Herein, our findings indicate that the S-palmitoylation of FAK is crucial for both its membrane localization and activation.

**Methods:**

The palmitoylation of FAK in U251 and T98G cells was assessed by an acyl-PEG exchange (APE) assay and a metabolic incorporation assay. Cellular palmitoylation was inhibited using 2-bromopalmitate, and the palmitoylation status and cellular localization of FAK were determined. A metabolic incorporation assay was used to identify the potential palmitoyl acyltransferase and the palmitoylation site of FAK. Cell Counting Kit-8 (CCK8) assays, colony formation assays, and Transwell assays were conducted to assess the impact of ZDHHC5 in GBM. Additionally, intracranial GBM xenografts were utilized to investigate the effects of genetically silencing ZDHHC5 on tumor growth.

**Results:**

Inhibiting FAK palmitoylation leads to its redistribution from the membrane to the cytoplasm and a decrease in its phosphorylation. Moreover, ZDHHC5, a protein-acyl-transferase (PAT), catalyzes this key modification of FAK at C456. Knockdown of ZDHHC5 abrogates the S-palmitoylation and membrane distribution of FAK and impairs cell proliferation, invasion, and epithelial-mesenchymal transition (EMT). Taken together, our research reveals the crucial role of ZDHHC5 as a PAT responsible for FAK S-palmitoylation, membrane localization, and activation.

**Conclusions:**

These results imply that targeting the ZDHHC5/FAK axis has the potential to be a promising strategy for therapeutic interventions for glioblastoma (GBM).

Video Abstract

**Supplementary Information:**

The online version contains supplementary material available at 10.1186/s12964-023-01366-z.

## Background

GBM is the most common primary malignant and lethal tumor in the central nervous system [1, 2]. With an annual incidence of approximately 5.26 per 100,000 population [3], it accounts for approximately 80% of intracranial cancers. Currently, the main treatments for GBM are surgery, radiation therapy, and chemotherapy [4]. The substantial genetic variability of this disease results in resistance to conventional treatments, despite recent improvements in these therapies [5]. Thus, there is an urgent need to uncover the mechanisms underlying the progression of GBM to develop more effective therapeutic strategies.

In the process of epithelial-mesenchymal transition (EMT), epithelial cells undergo a transformation into migratory and invaded cells, which play a vital role in tumor the invasion and progression [6–9]. In glioma, EMT plays a crucial role in initiation of angiogenesis, resistance to cell death, and stimulation of invasion and metastasis [10]. During EMT, glioma cells transition into a mesenchymal phenotype, acquiring enhanced invasion and migration capabilities. This transition is characterized by the expression of EMT markers including N-cadherin, β-catenin, Snail and Slug, and the repression of epithelial markers including E-cadherin [11–13]. In addition, several mesenchymal gene sets have been found to exhibit obvious correlation with the radioresistance profile which indicates that EMT plays a crucial role in radioresistance in GBM [14]. Accumulating evidence indicates that a highly sensitive molecular regulator of EMT could serve as a promising marker for glioma.

FAK is a member of the nonreceptor tyrosine kinase family and plays a crucial role in the regulation of cell migration [15]. Integrin clustering causes FAK to be recruited to newly generated focal adhesion sites, and various downstream effectors are phosphorylated after FAK activation, resulting in angiogenesis, cell migration, and cell proliferation [16–19]. Moreover, increased FAK protein levels have been reported in cancers derived from many tissues and in tumor cell lines, while FAK expression is undetectable or low in benign neoplasms and normal tissues [20–25]*.* While recent studies have confirmed FAK's involvement in tumor invasion and metastasis, the underlying mechanism by which FAK influences tumor cell EMT remains unclear.

Protein acyltransferases are distinguished by a conserved catalytic domain known as the Asp-His-His-Cys (DHHC) domain and control protein palmitoylation [26, 27], a prevalent and reversible lipid posttranslational modification [28], that involves the addition of a 16-carbon palmitoyl group to a cysteine residue through a thioester linkage [29]. Accumulating investigations have demonstrated that DHHC proteins and their substrates are essential for tumor cell EMT, migration, and invasion [30–33]. ZDHHC19 exhibits elevated expression in malignant cervical cancer tissues, consequently driving cervical cancer cells EMT, migration, and invasion [33]. Moreover, ZDHHC1 overexpression was found lead to increased expression of epithelial markers like E-cadherin and Occludin, while reducing the expression of mesenchymal markers such as Vimentin and N-cadherin, indicating that ZDHHC1 acts as a tumor suppressor by inhibiting EMT in human cancers. A recent study revealed that ZDHHC5 increases the self-renewal capability of glioblastoma stem cells (GSCs), thereby actively contributing to the tumorigenicity of glioma cells [31]. However, the mechanism by which ZDHHC5 affects the EMT process in GBM remains unknown.

In this study, we observed the palmitoylation of FAK in GBM cell lines, and this modification was found to play a crucial role in the membrane localization of FAK. Through additional screening assays for palmitoyltransferases (PATs), we found that ZDHHC5 is the key enzyme responsible for the S-palmitoylation of FAK. Specifically, ZDHHC5 palmitoylates FAK at Cys456, ensuring its membrane localization and contributing to the induction of epithelial-mesenchymal transition (EMT), which promotes the development of GBM.

## Methods

### Cell culture

Human embryonic kidney cells (HEK293T), U251 cells, and T98G cells were obtained from the American Type Culture Collection (ATCC) (Manassas, VA). These cell lines were cultured and maintained in Dulbecco's modified Eagle's medium (DMEM) (Life Technologies), containing 10% foetal bovine serum (FBS) (Thermo/HyClone, Waltham, MA). Cell cultures were maintained at 37 °C in a 5% CO_2_ atmosphere and regularly tested for mycoplasma contamination.

### Antibodies and reagents

Antibodies specific for the following proteins and tags were used: FAK (Abcam, #ab76496), ZDHHC5 (CST, #79842), β-actin (Proteintech, #81115–1-RR), Ubiquitin (CST, #20326), Flag (Abcam, #ab205606), Myc (Abcam, #ab9106), HA (CST, #3724), phospho-FAK (Abcam, #ab81298), Paxillin (Abcam, #ab32084), phospho-Paxillin (Abcam, #ab109547), AKT (Abcam, #ab8805), phospho-AKT (Abcam, #ab81283), ERK (Abcam, #ab184699), phospho-ERK (Abcam, #ab201015), α-Tubulin (Proteintech, #11,224–1-AP), ATP1A1 (Abcam, #ab7671), E-cadherin (Abcam, #ab219332), N-cadherin (Abcam, #ab18203), snail (Sigma-Aldrich, #SAB5700703), slug (Sigma-Aldrich, #SAB5700672), and β-catenin (Abcam, #ab32572).

The following reagents were used: MG132 (Beyotime, #S1748), cycloheximide (Sigma-Aldrich, #66–81-9), 2-bromopalmitate (Sigma-Aldrich, #238442), palmostatin B (Sigma-Aldrich, #178501), DMSO (Beyotime, #ST038), MeO-PEG-Mal (Sigma-Aldrich, #63187), hydroxylamine (Sigma-Aldrich, #159417), N-ethylmaleimide (NEM) (Sigma-Aldrich, #04260), and TCEP (Sigma-Aldrich, #646547).

### Plasmid construction

The pcDNA3.1 vector was used for construction of the Flag-FAK and Myc-ZDHHC5 plasmids. All mutants were generated using the QuickChangeII Site-Directed Mutagenesis Kit (Agilent) following the manufacturer's instructions. Plasmids containing HA-ubiquitin were acquired from Addgene (#18712).

### shRNAs, siRNAs, and transfection

Lentiviral particles containing a shRNA targeting ZDHHC5, or a scrambled nontargeting control sequence were obtained from Invitrogen (Carlsbad, CA, USA). The ZDHHC5 shRNA sequences were as follows: shRNA#1, 5'-CACCGGAAGCATTAGTGTTGACTGGCGAACCAGTCAACACTAATGCTTCC3'; and shRNA#2, 5'-CACCGAAATCAAGCCTGACGAAGTTCGAAAACTTCGTCAGGCTTGATTTC-3'. The FAK shRNA sequences were as follows: shRNA#1, 5'-GATGTTGGTTTAAAGCGATTT3'; and shRNA#2, 5'-CCGATTGGAAACCAACATATA3'. Table S1 lists the siRNAs that were used in this study to target ZDHHCs. Cells were transfected with the indicated plasmids, shRNAs, and siRNAs using Lipofectamine 3000 (Invitrogen), according to the manufacturer's instructions.

### Immunoblotting (IB) and immunoprecipitation (IP)

Cells were lysed in RIPA buffer containing protease inhibitors (Thermo Scientific, #87785) and phosphatase inhibitors (Sigma-Aldrich, #P0044). The lysates were heated for 10 min at 100 °C to denature proteins. After SDS-PAGE, the proteins were transferred onto PVDF membranes (Millipore). Then the membranes were incubated with the indicated primary antibodies at 4 °C overnight, subjected to three washes with TBST buffer, and subsequently incubated with the HRP-conjugated secondary antibody. Immunoblots were visualized with chemiluminescence reagents (Thermo Fisher, #34580). For immunoprecipitation, cells were lysed in lysis buffer (Beyotime, #P0013) supplemented with protease inhibitors and phosphatase inhibitors. The cell lysates were precleared by incubation with protein A/G agarose (Thermo Fisher Scientific) for 1 h at 4 °C. After the preclearing step, the lysates were subjected to immunoprecipitated overnight at 4 °C using the indicated antibodies, and then further incubated with protein A/G agarose for an additional 2 h at 4 °C. Following three washes with PBS buffer, the immunocomplexes were separated via SDS-PAGE, transferred onto PVDF membranes, and analysed by IB.

### Protein purification and GST pull-down assay

The GST fusion protein GST-FAK was produced by cloning FAK cDNA in-frame into the pGEX6p-1 vector for the GST pull-down assay. Cell lysates were treated with glutathione beads (Sigma, #G0924) overnight at 4 °C after centrifugation. GST-FAK was purified according to the manufacturer’s protocol. Flag-tagged proteins were purified using anti-Flag magnetic beads (Beyotime, #P2115,) after Myc-ZDHHC5 and its mutant were transfected into HEK293T cells. The recombinant proteins were stored at -80 °C and analysed by SDS-PAGE. For the GST pull-down assay, GST and the GST-FAK fusion protei were immobilized onto glutathione-sepharose beads (Sigma-Aldrich) and incubated with U251 cell lysates overnight. Following three washes, the bound proteins were eluted using sodium dodecyl sulfate (SDS) loading buffer and subjected to analysis by SDS-PAGE.

### In vivo ubiquitination assay

We utilized denaturing immunoprecipitation (d-IP) to perform an in vivo experiment aimed at examining FAK ubiquitination. Cells were transfected with the indicated plasmids along with HA-ubiquitin for a duration of 48 h. Then the cells were exposed to MG132, a proteasome inhibitor, at a concentration of 20 μM for a duration of 6 h. After treatment, the cells were lysed with a denaturing lysis buffer containing 62.5 mM Tris–HCl (pH 6.8), 1.5% β-mercaptoethanol, 10% glycerol, and 2% SDS. Cell lysates were subjected to IP using the indicated antibodies and analysed by IB with an anti-HA antibody.

### Metabolic incorporation assay

Cells were incubated with 100 μM palmitic acid azide or DMSO in serum-free DMEM for 4 h at 37 °C. Subsequently, the cells were washed with PBS and lysed in lysis buffer (50 mM TEA-HCl (pH 7.5), 150 mM NaCl, 0.1% Triton X-100, 0.2% SDS, protease inhibitors). Proteins in the lysates then underwent click reaction with biotin-azide. Proteins were precipitated with 9 volumes of 100% methanol for 2 h at -80 °C and retrieved through centrifugation at 15,000 × g for 10 min. The resulting pellets were dissolved in 100 ml suspension buffer (50 mM Tris–HCl (pH 7.4), 150 mM NaCl, 5 mM EDTA, 2% SDS) and subsequently diluted tenfold using immunoprecipitation buffer (50 mM Tris–HCl, pH 7.4, 150 mM NaCl, 5 mM EDTA, 0.5% NP40). Biotinylated proteins were isolated using streptavidin agarose and washed five times with wash buffer (50 mM Tris (pH 7.5), 0.1% SDS), and the samples were then analysed by IB.

### Acyl-PEG exchange (APE) assay

The APE assay was performed according to a previously reported method [34]. The collected cells were lysed in lysis buffer (50 mM TEA-HCl (pH 7.4), 150 mM NaCl, 1% Triton X-100, and 2% SDS) with protease and phosphatase inhibitors and then incubated with 20 mM TCEP at 100 rpm for 1 h at 55 °C. Free cysteine residues were blocked by incubation with 50 mM NEM at 55 °C and 100 rpm for 3 h. After protein precipitation using methanol/chloroform, the mixture was incubated with NH2OH at 37 °C for 1 h to break the cysteine-palmitoyl thioester linkages. Proteins were precipitated with methanol-chlorofor, and then incubated with 2 mM PEG at room temperature. After 1 h, proteins were precipitated again with methanol-chloroform, and were then ree-suspended in loading buffer and boiled for 5 min at 100 °C prior to western blotting analysis.

### Subcellular fractionation

Cells were harvested and suspended in subcellular fractionation buffer (250 mM sucrose, 10 mM KCl, 1.5 mM MgCl_2_, 20 mM HEPES (pH 7.4), 1 mM EDTA, 1 mM DTT, and 1 mM EGT) supplemented with protease inhibitor cocktail. Each was subjected to centrifugation at 1000 × g for 5 min to obtain the postnuclear supernatant. This supernatant was then further centrifuged at 6000 × g for 5 min to sparate the mitochondrial fraction. The supernatant resulting from centrifugation at 6000 × g was subject to another round of centrifugation at 20,000 × g for 2 h to separate the membrane fraction (pellet). The supernatant remaining after centrifugation at 20,000 × g was designated the cytosolic fraction.

### CCK-8 assay

For the CCK8 assay, approximately 2000 tumor cells were plated in each well of a 96-well plate. In accordance with the aforementioned conditions, 10 µL of CCK-8 reagent (Beyotime, #C0037) was added to each well. A microplate reader was used to calculate the absorbance at 450 nm. At least three replicates were established for each group.

### Colony formation

For the colony formation assay, approximately 300 tumor cells were seeded into each well of a 6-well plate and allowed to grow for a period of 14 days. After the incubation period, the cells were fixed with 4% formaldehyde for 10 min. Subsequently, the cells were stained with crystal violet (Servicebio, #G1014) at room temperature for 30 min before the calculation.

### Invasion, migration, and wound healing assays

Invasion and migration assays were carried out using Corning Matrigel invasion chambers. U251 and T98G cells were seeded into a 24-well plate, and chambers containing a membrane with or without a Matrigel coating were inserted. The upper compartments were filled with 100 µL serum-free medium, the lower compartments were filled with 500 µL of DMEM containing 30% FBS, the plates were incubation at 37 °C for a duration of 24 h. The cells in the lower compartments were fixed and stained with Giemsa after the floating cells had been removed from the incubation chamber. Images of cells were analysed using a Nikon inverted microscope.

In the wound healing assay, cells were seeded into a 6-well plate and cultured to approximately 90% confluence. Subsequently, the cells were detached from the plate surface by gently scraping with a 10-μl pipette tip and maintained in serum-free DMEM after being washed with PBS. Photographs were taken at 0 h and 24 h after wounding. Cell migration was quantified by calculating the percentage of the gap width at the indicated time relative to the gap width at 0 h. Statistical analysis was conducted using Image-Pro Plus 6.0 software, and the results are presented as the average values obtained from three independent experiments.

### Immunofluorescence (IF) staining

Cells were plated in confocal dishes and then fixed with paraformaldehyde for 20 min. The fixed cells were washed twice with PBS, permeabilized and blocked with a solution containing 0.1% saponin, 5% BSA, and PBS for 30 min. The cells were incubated with the indicated antibodies overnight at 4 °C in the dark. After three washes with PBS, the cells were incubated with a secondary antibody at room temperature in the dark for 1 h. The confocal dishes were then mounted with DAPI-Aqueous (Abcam, #104,139). Subsequently, the images were acquired using laser confocal microscope and analysed using LAS X software.

### Xenograft assay

T98G or U251 cells (5 × 10^5^) in 5 μl DMEM were intracranially injected into female 5-week-old athymic nude mice (ten mice per group) obtained from Vital River (Beijing, China). The occurrence of neurological signs in each mouse was monitored daily. Bioluminescence imaging was conducted with an IVIS Lumina Imaging System, which allowed for visualization and tracking of tumor growth in the mice over time. Between 2 and 12 weeks after implantation, the mice were euthanized humanely, and their brains were removed, paraffin-embedded, and subjected to immunohistochemical staining. All animal research conducted in this study was approved by the Institutional Animal Care and Use Committee (IACUC) of Nanjing Medical University, following the necessary regulations. The research was conducted in accordance with approved protocols and guidelines, with approval number 2210021.

### Human tissues and immunohistochemical (IHC) staining

Shanghai Outdo Biotech provided human glioma tissues. All patients gave their informed consent, and the Biotech Ethics Committee of Shanghai Outdo (Shanghai, China) authorized all use of human tissues in this study.

Servicebio Technology (Wuhan, China) used the indicated antibodies to conduct IHC analysis. Through the use of Case Viewer and 3DHISTECH QuantCenter 2.1 software, staining was evaluated. Tumor samples collected from mice were fixed and prepared for IHC staining. The percentage of positively stained cells was scored based on the following criteria: 0 (0%), 1 (1%–24%), 2 (25%–49%;), 3 (50%–74%), and 4 (75%–100%). The following criteria were used to determine the staining intensity scores: 0, negative; 1, weak; 2, moderate; and 3, strong staining. Finally, the two scores were multiplied to obtain the final score, which was then categorized into two grades: low expression (score: 0–4); and high expression (score:5–12).

### Statistical analysis

GraphPad Prism software (Version 7.0) was used to conduct statistical analysis. The means and standard deviations between the values for the control and experimental groups were analysed using two-tailed unpaired or paired Student's t test. The Kaplan–Meier method was used to evaluate the survival rate. Data are expressed as the means ± SDs and *P* values of 0.05 or less were considered to indicate a significant difference.

## Results

### S-palmitoylation maintains the membrane localization of FAK

**Fig. 1:**
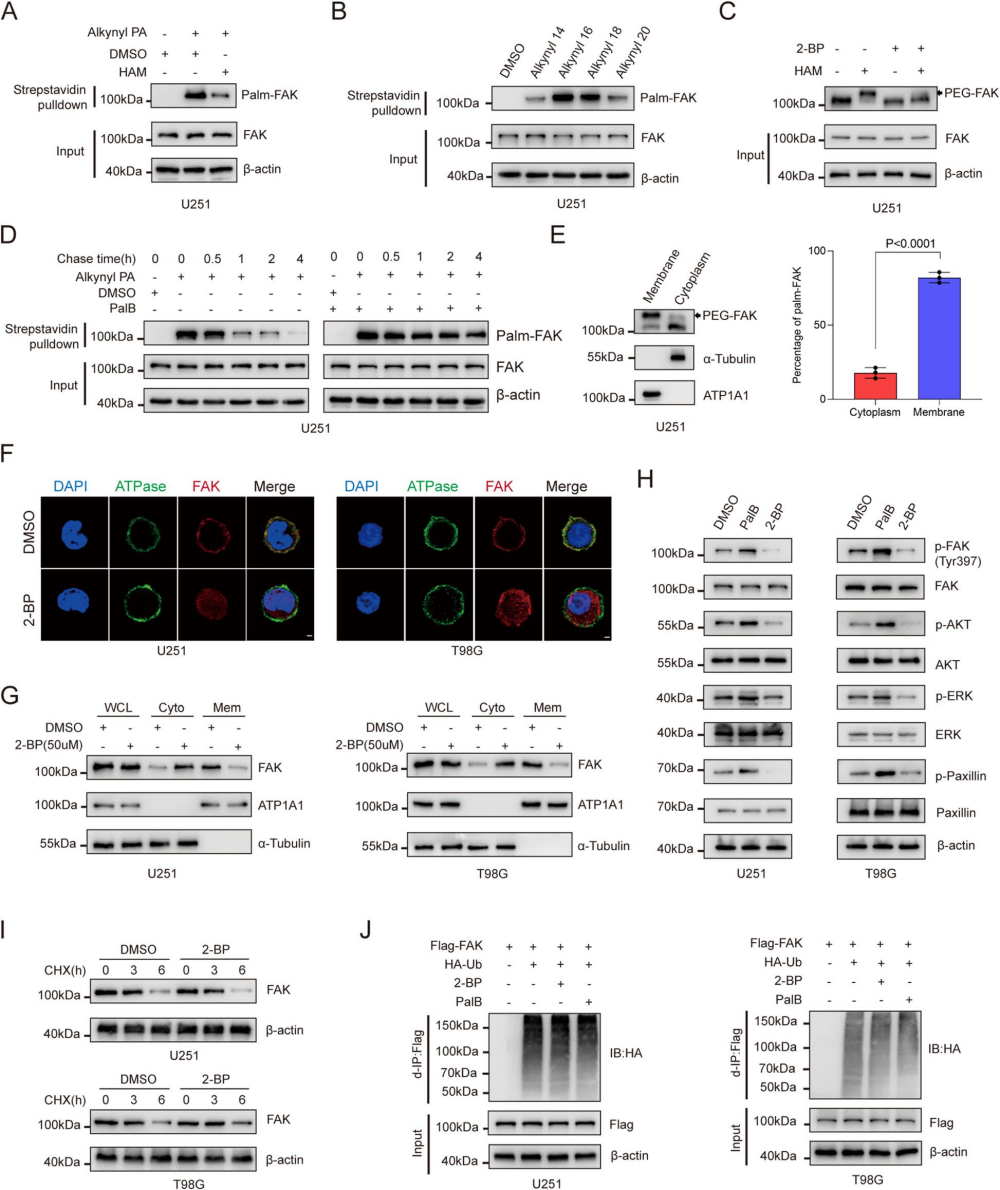
S-palmitoylation maintains the membrane localization of FAK (**A**) FAK palmitoylation was examined in lysates obtained from U251 cells that were metabolically treated with a palmitoylation probe (50 μM alkynyl palmitic acid [PA]) for a duration of 4 h. The analysis was carried out through click reaction and streptavidin bead pulldown, both in the absence and presence of hydroxylamine (HAM). The subsequent immunoblotting (IB) was performed using the indicated antibodies. **B** FAK fatty acylation levels were investigated using different chemical reporters of fatty acylation, ranging from Alk-C14 to Alk-C20. The analysis involved the use of streptavidin bead pulldown to isolate acylated FAK, followed by IB. **C** APE assays were conducted to examine the levels of FAK palmitoylation in U251 cells following treatment with 50 μM 2-BP, both in the absence and presence of HAM. **D** FAK palmitoylation studies were conducted in U251 cells that were metabolically labeled with 50 µM alkynyl PA for a duration of 4 h. The cells were treated either in the absence or presence of PalB (5 μM). After the specified time period, the cells were collected for further analysis of FAK palmitoylation. **E** FAK palmitoylation was analyzed using the APE assay after fractionation to distinguish between the cytoplasmic and membrane fractions. ATP1A1 and α-tubulin were used as controls for the membrane and cytoplasmic fractions, respectively. Quantification of FAK palmitoylation percentage in the cytoplasm and membrane in APE assays. All the data are presented as the mean ± SD, *n* = three independent experiments, and two-tailed Student’s t test. **F** U251 and T98G cells were treated with dimethyl sulfoxide (DMSO) or 50 μM 2-BP for 8 h, and the cellular localization of endogenous FAK was visualized using immunofluorescence staining. Scale bar, 1 μm. **G** U251 and T98G cells were subjected to treatment with either DMSO or 50 μM 2-BP for 8 h. The levels of FAK in the membrane and cytoplasmic fractions were evaluated using IB with the indicated antibodies. ATP1A1 and α-tubulin were used as membranal and cytoplasmic fraction controls, respectively. WCL refers to the whole-cell lysate, Mem refers to the membrane, and Cyto refers to the cytoplasm. **H** U251 and T98G cells were treated with DMSO, 50 μM 2-BP, or 2.5 μM PalB. Then, IB was performed for the indicated proteins. **I** U251 and T98G cells were pretreated with 2-BP or DMSO as a control and then incubated with cycloheximide (CHX). Immunoblotting was used to analyze the FAK and β-actin levels at the specified time points. **J** U251 and T98G cells were cotransfected with HA-Ub and treated with DMSO, 50 μM 2-BP, or 5 μM PalB for 8 h. U251 and T98G cells were subjected to IP-IB and IB for the indicated proteins

To investigate the palmitoylation of FAK, we conducted a metabolic incorporation assay. Our findings confirmed that endogenous FAK undergoes S-palmitoylation, which occurs through the formation of thioester bonds that can be cleaved by treatment with hydroxylamine (HAM) (Fig. 1A and Figure S1A). Additionally, FAK can be labelled with high efficiency using Alk-C16 or Alk-C18 (Fig. 1B). However, it exhibits lower labelling efficiency with C14 or C20 chain length probes (Fig. 1B and Figure S1B), suggesting that FAK is predominantly modified with palmitoyl (C16) or stearoyl (C18) groups, while other chain lengths may have lower affinity or specificity for FAK. To further explore the palmitoylation levels of FAK, we used an acyl-PEG exchange (APE) assay. Upon treatment with 2-bromopalmitate (2-BP), a paninhibitor of PATs, the palmitoylation level of endogenous FAK was significantly decreased (Fig. 1C and Figure S1C). Moreover, in pulse-chase experiments, we observed that the turnover half-life of FAK palmitoylation was approximately one hour, while the overall protein level remained unchanged, indicating that FAK S-palmitoylation is a dynamic process (Fig. 1D and Figure S1D). Interestingly, the acyl-PEG exchange (APE) assay showed that membrane FAK was substantially palmitoylated (~ 80%) compared to cytoplasmic FAK (~ 20%) (Fig. 1E and Figure S1E), suggesting a direct correlation between the palmitoylation and membrane localization of FAK. To further verify this hypothesis, we monitored its cellular distribution following 2-BP treatment via immunofluorescence (IF) staining and found that FAK was redistributed from the membrane to the cytoplasm upon 2-BP treatment (Fig. 1F). Consistently, immunoblot analysis confirmed that the membrane localization of endogenous FAK was significantly reduced upon treatment with 2-BP in U251 and T98G cells, whereas the total level of FAK protein remained unchanged (Fig. 1G).

It is established that membrane-bound FAK is primed for activation [35]. Upon the association of FAK with the membrane, FAK autoinhibition is released, leading to efficient autophosphorylation at Tyr397 [35, 36], which consequently activates downstream pathways of FAK, including the PI3K/AKT, ERK, and Paxillin signalling pathways [37–39]. Given the above results, we next determined whether palmitoylation is required for FAK activation. U251 and T98G cells treated with palmostatin B (PalB), an inhibitor of deacylating enzymes, exhibited elevated levels of phosphorylated FAK, AKT, ERK, and Paxillin (Fig. 1H). In contrast, inhibition of FAK palmitoylation with 2-BP dramatically decreased the levels of p-FAK, p-AKT, p-ERK, and p-Paxillin (Fig. 1H). Additionally, we found that neither 2-BP treatment nor PalB treatment altered the decay rate or polyubiquitination level of FAK (Fig. 1I and J). Taken together, these data suggest that FAK undergoes S-palmitoylation, which is required for maintaining its membrane localization.

### ZDHHC5 regulates FAK S-palmitoylation by directly binding to FAK

**Fig. 2:**
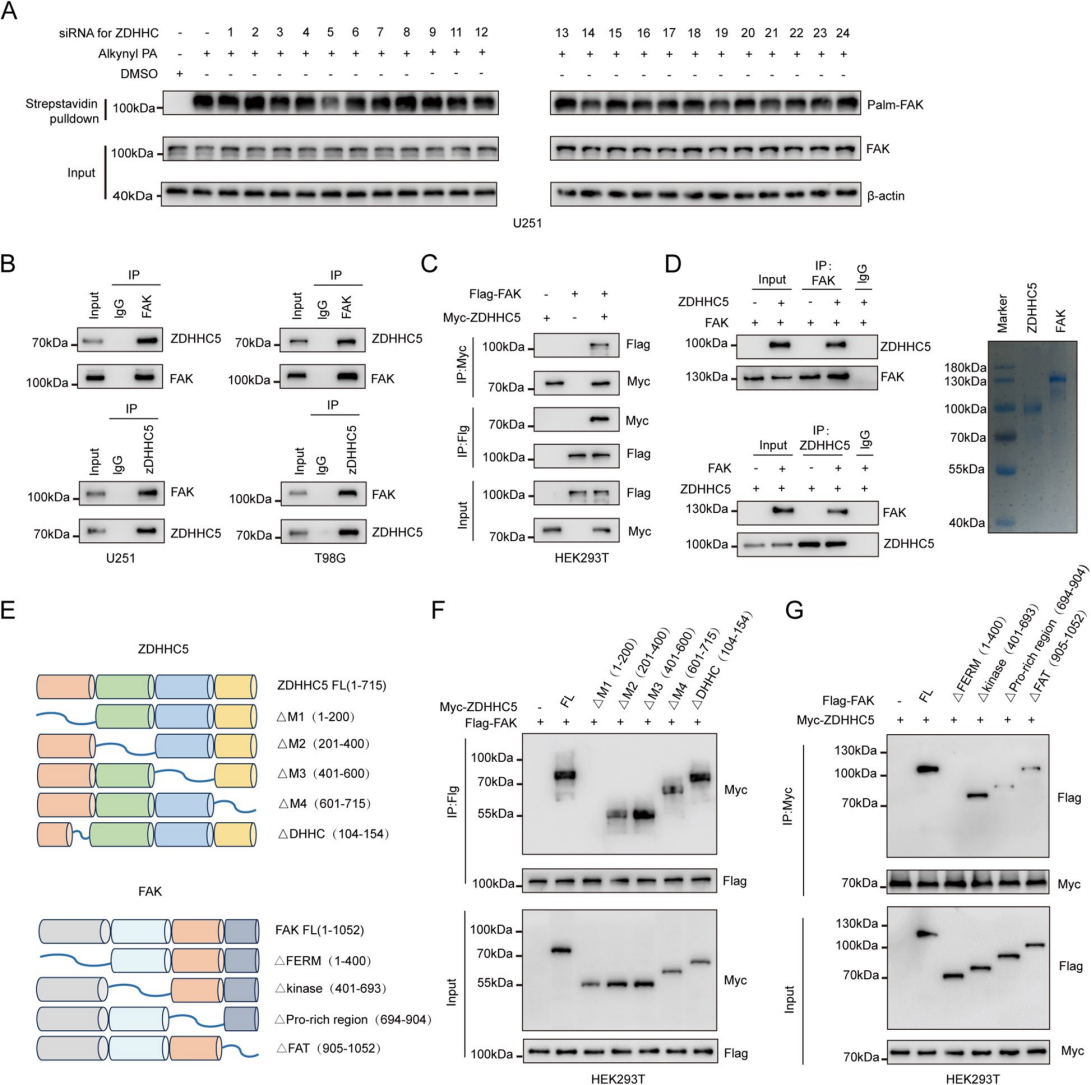
ZDHHC5 regulates FAK S-palmitoylation by directly binding to FAK (**A**) U251 cells infected with siRNAs targeting different zDHHC proteins. The cells were metabolically labeled with alkynyl PA (50 µM) for 4 h. FAK palmitoylation levels were assessed by click reaction and streptavidin bead pulldown, followed by IB. **B** Cell lysates from U251 and T98G cells were analyzed by IP using antibodies against FAK and ZDHHC5 and then subjected to IB analysis. IgG was used as the isotype control. **C** HEK293T cells transfected with Flag-FAK and/or Myc-ZDHHC5 were subjected to IP-IB and IB using the indicated antibodies. **D** In vitro protein binding assays with purified ZDHHC5 and FAK protein. The purities of ZDHHC5 and FAK were examined by SDS-PAGE and Coomassie Blue Staining. **E** Schematic representations of ZDHHC5, FAK, and their shortened mutants. **F** HEK293T cells were cotransfected with Flag-FAK and FL Myc-ZDHHC5 or its deletion mutants. The cell lysates were subjected to Flag bead pulldown, followed by IB using specific antibodies. **G** HEK293T cells were cotransfected with Myc-ZDHHC5 and FL Flag-FAK or its deletion mutants. Cell lysates were analyzed by Flag bead pulldown, followed by IB using the indicated antibodies

To recognize the potential palmitoyl acyltransferase targeting FAK, a series of siRNAs designed to target PATs were introduced into U251 cells and we found that knockdown of ZDHHC5 but not other PATs almost completely abolished the S-palmitoylation of FAK, suggesting that ZDHHC5 is the major PAT that mediates FAK palmitoylation in GBM cells (Fig. 2A). Moreover, coimmunoprecipitation (Co-IP) assays revealed endogenous and exogenous interactions between ZDHHC5 and FAK (Fig. 2B and C). Furthermore, the GST pull-down assay and in vitro binding experiment showed that both WT ZDHHC5 and its catalytically inactive C134S mutant could interact with FAK (Fig. 2D and Figure S2A), which demonstrated a direct interaction between ZDHHC5 and FAK. To identify the regions required for this interaction, we generated a series of truncation mutants of Myc-ZDHHC5 and Flag-FAK (Fig. 2E). We found that ZDHHC5 interacts with FAK through an N-terminal sequence (amino acids 1 to 200). Remarkably, ZDHHC5 ΔDHHC had the same affinity for FAK as the full-length ZDHHC5 protein, suggesting that the ZDHHC5-FAK interaction is not dependent on the PAT activity of ZDHHC5 (Fig. 2F). Accordingly, we generated four Flag-FAK truncation mutants lacking either the FERM domain, kinase domain, Pro-rich region, or FAT domain. The coimmunoprecipitation assay revealed that Myc-ZDHHC5 could be immunoprecipitated by the Δkinase, ΔPro-rich region, and ΔFAT Flag-FAK truncation mutants. However, when FAK lacked the FERM domain, the interaction between ZDHHC5 and FAK was significantly diminished (Fig. 2G). These results indicate that ZDHHC5 binds directly to FAK.

### ZDHHC5 maintains FAK membrane localization and activation via S-palmitoylation

**Fig. 3:**
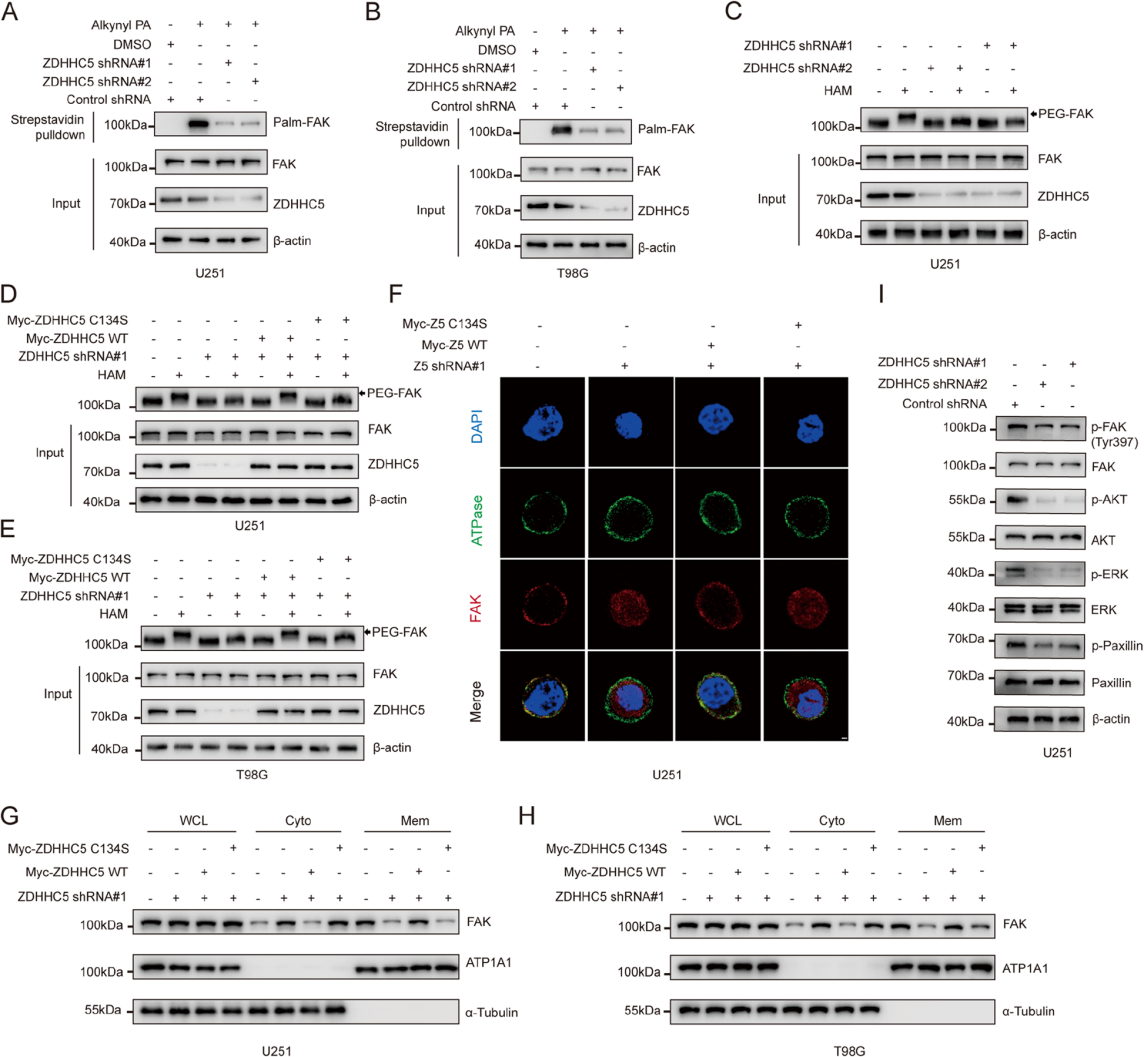
ZDHHC5 maintains FAK membrane localization and activation via S-palmitoylation (**A**) and (**B**) U251 and T98G cells were transduced with lentiviruses carrying either control shRNA or ZDHHC5 shRNAs. Subsequently, the cells were metabolically labeled with 50 μM alkynyl PA for a duration of 4 h. The levels of FAK palmitoylation were analyzed by performing a click reaction and streptavidin bead pulldown, followed by IB. **C** An APE assay was performed to analyze FAK palmitoylation in ZDHHC5-knockdown U251 cells. **D** and **E** ZDHHC5-knockdown U251 and T98G cells were rescued with Myc-ZDHHC5 WT or ZDHHC5 C134S. FAK palmitoylation levels were analyzed by APE assay. **F** ZDHHC5-knockdown U251 cells were rescued with Myc-ZDHHC5 WT or ZDHHC5 C134S, and endogenous FAK cellular localization was visualized by immunofluorescence staining using antibodies against FAK. Scale bar, 1 μm. Z5, ZDHHC5 (**G**), and (**H**) ZDHHC5-knockdown U251 cells were rescued with Myc-ZDHHC5 WT or ZDHHC5 C134S. The levels of FAK in the membrane and cytoplasmic fractions were evaluated using IB with the indicated antibodies. **I** U251 cells were infected with lentiviruses expressing control shRNA or ZDHHC5 shRNAs and then subjected to IB for the indicated proteins

To further confirm that ZDHHC5 regulates the S-palmitoylation of FAK, we performed a metabolic incorporation assay and an APE assay. Silencing ZDHHC5 abrogated FAK S-palmitoylation (Fig. 3A-C and Figure S3A), and this effect was reversed upon introduction of WT ZDHHC5, but not the ZDHHC5 C134S mutant (Fig. 3D and E). These findings indicate that the enzymatic activity of ZDHHC5 is crucial for the S-palmitoylation of FAK. Moreover, immunofluorescence analyses confirmed that ZDHHC5 knockdown by shRNA significantly inhibited the association of FAK with the membrane and that this association was restored by introduction of WT ZDHHC5, but not the C134S mutant (Fig. 3F and Figure S3B). The findings were additionally validated through cellular fractionation assays (Fig. 3G and H). Consistently, silencing ZDHHC5 significantly decreased the phosphorylation of FAK and the activation of downstream signalling pathways (Fig. 3I and S3C). Taken together, these findings reveal that ZDHHC5 facilitates the S-palmitoylation of FAK, maintains its membrane localization and promotes its activation.

### FAK is palmitoylated at Cys456

**Fig. 4:**
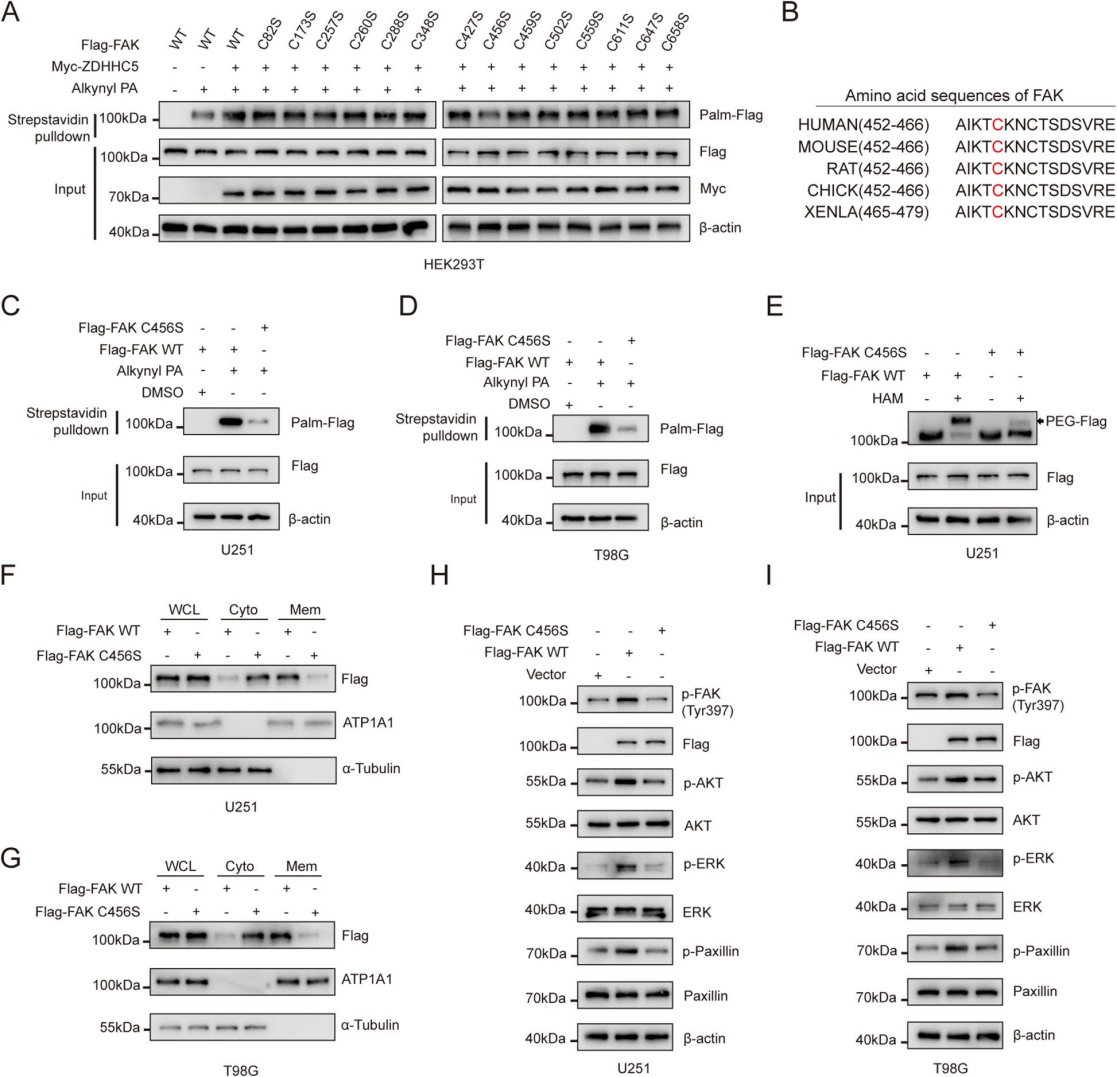
FAK is palmitoylated at Cys456 (**A**) U251 cells were cotransfected with Flag-FAK WT or the indicated mutants and/or Myc-ZDHHC5. The cells were metabolically labeled with 50 µM alkynyl PA for 4 h. The levels of FAK palmitoylation were analyzed by performing a click reaction and streptavidin bead pulldown, followed by IB. **B** Amino acid sequences around the cysteine 456 residue of the FAK protein across different species. **C** and **D** U251 and T98G cells were transfected with Flag-FAK WT or Flag-FAK C456S and then metabolically labeled with 50 M alkynyl PA for 4 h. FAK palmitoylation levels were assessed by click reaction and streptavidin bead pulldown, followed by IB. **E** U251 and T98G cells were transfected with Flag-FAK WT or Flag-FAK C456S. The APE assay was conducted to analyze FAK palmitoylation in U251 cells with the specified modifications. **F** and **G** U251 and T98G cells were transfected with Flag-FAK WT or Flag-FAK C456S. The levels of FAK in the membrane and cytoplasmic fractions were evaluated using IB with the indicated antibodies. **H** and **I** U251 and T98G cells were transfected with Flag-FAK WT or Flag-FAK C456S. Then, IB was performed with the indicated antibodies

To investigate the site of FAK palmitoylation mediated by ZDHHC5, we mutated all cysteine (C) residues in FAK to serine (S) and found that the overexpression of ZDHHC5 had no impact on the palmitoylation level of the FAK C456S mutant (Fig. 4A), suggesting that ZDHHC5 mediates the palmitoylation of FAK at C456. Notably, the amino acid sequence surrounding C456 exhibits marked species conservation (Fig. 4B). Consistent with this finding, the Flag-FAK C456S mutant displayed decreased a level of palmitoylation compared to that of Flag-FAK WT in U251 and T98G cells, as determined by the metabolic incorporation assay and APE assay (Fig. 4C-E and Figure S4A).

We next investigated whether palmitoylation of FAK at C456 regulates its membrane localization and activation. The cellular fractionation assay confirmed that the FAK C456S mutant predominantly localized in the cytoplasm compared to FAK WT, resembling the effects observed following treatment with 2-BP (Fig. 4F and G). Moreover, we examined the activation of FAK and found that overexpression of FAK WT but not the FAK C456S mutant promoted the phosphorylation of FAK and the activation of downstream signalling pathways (Fig. 4H and I). This finding suggests that ZDHHC5 mediates FAK S-palmitoylation at C456, which promotes FAK membrane localization and activation.

### ZDHHC5-mediated FAK S-palmitoylation promotes cell proliferation, cell invasion and EMT in vitro

**Fig. 5:**
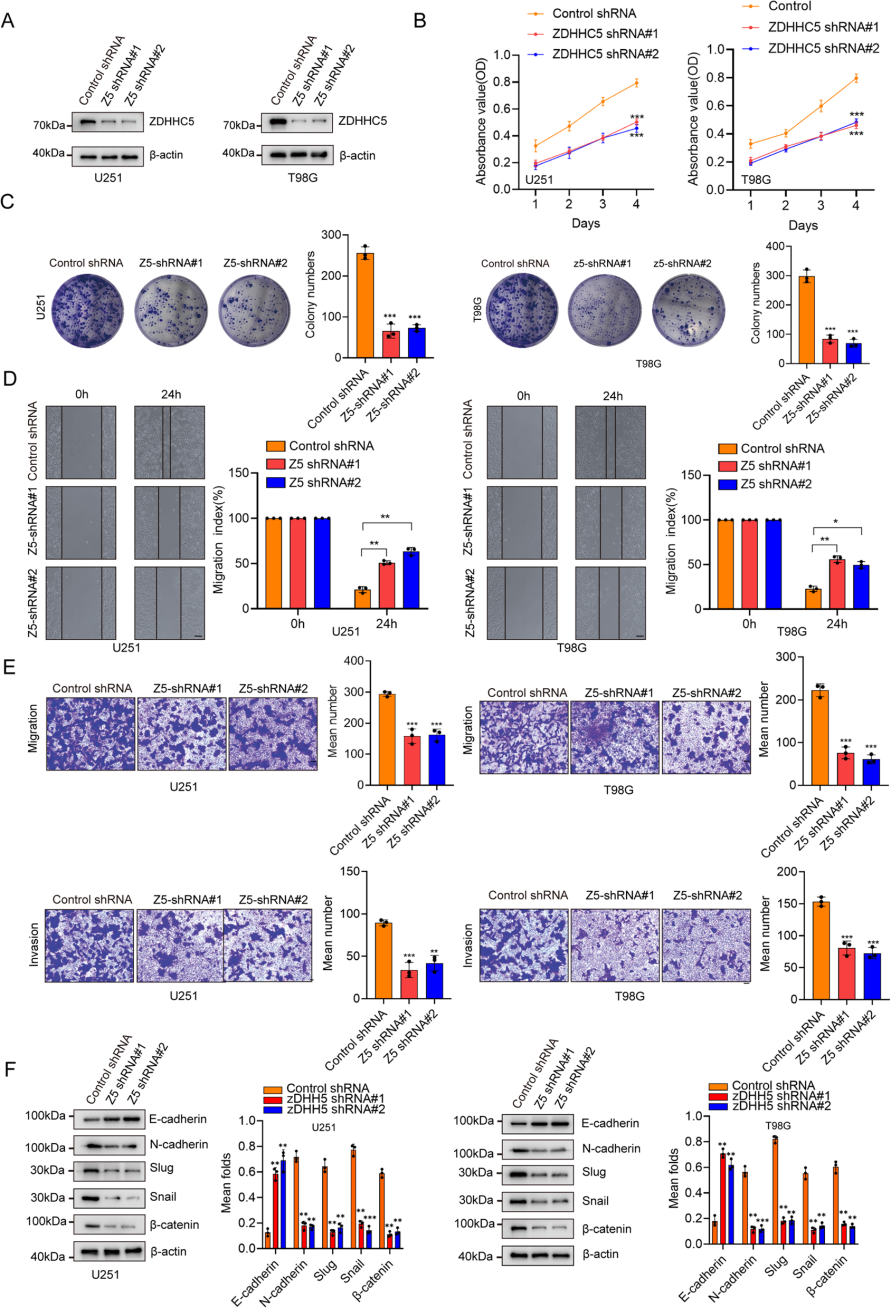
ZDHHC5-mediated FAK S-palmitoylation promotes cell proliferation, cell invasion and EMT in vitro. **A** IB for ZDHHC5 in U251 and T98G cells transfected with ZDHHC5 shRNAs or shRNA control. **B** CCK8 assays of U251 and T98G cells transfected with ZDHHC5 shRNAs or shRNA control. **C** Colony formation assay of U251 and T98G cells transfected with ZDHHC5 shRNAs or shRNA control. **D** Wound-healing assay of ZDHHC5 shRNAs or shRNA control. scale bars: 25 μm. **E** Invasion of U251 and T98G cells transfected with ZDHHC5 shRNAs or shRNA control; scale bars: 100 μm. (F) IB for the indicated proteins in U251 and T98G cells transfected with ZDHHC5 shRNAs or shRNA control. Data are represented as the mean ± SD (*n* = 3). Statistical analysis was performed using Student’s t test, *p < 0.05; **p < 0.01; ***p < 0.001; ****p < 0.0001

To determine the role of ZDHHC5 in GBM, we silenced ZDHHC5 expression in U251 and T98G cells in U251 and T98G cells with ZDHHC5 knockdown (Fig. 5A). The results of CCK8 assays demonstrated that knockdown of ZDHHC5 significantly suppressed cell proliferation (Fig. 5B), which was further validated through colony formation assays (Fig. 5C). Subsequently, we investigated the influence of the ZDHHC5 shRNA on the migration and invasion capacities of U251 and T98G cells. The wound-healing assays showed that U251 and T98G cells with ZDHHC5 knockdown exhibited a significantly reduced wound closure area after 24 h compared to the negative control (NC) cells (Fig. 5D). The transwell assays also showed consistent results that knockdown of ZDHHC5 resulted in significant decreases in the percentages of migrated and invaded cells (Fig. 5E). Moreover, our findings revealed notable reductions in the expression levels of N-cadherin, snail, slug, and β-catenin, along with an increase in the E-cadherin level, in ZDHHC5-depleted U251 and T98G cells compared to the control cells (Fig. 5F). Remarkably, we found that these inhibitory effects of ZDHHC5 depletion were largely reversed by the overexpression of membrane-anchored and constitutively active mutants of FAK (CD2-FAK) [40, 41] but not by FAK C456S (Figure S5A-F). In addition, in a published dataset, CGGA, the mRNA level of ZDHHC5 was significantly higher in high-grade tumors (WHO III and WHO IV) than in low-grade tumors (WHO II) (Figure S5G). Kaplan–Meier survival analysis revealed that the mRNA level of ZDHHC5 had prognostic value for patient survival (Figure S5H).

To further investigate the role of ZDHHC5-mediated FAK S-palmitoylation in regulating cell proliferation, invasion and EMT, we knock down FAK in U251 and T98G cells and reintroduce Flag-FAK WT or FAK C456S mutant (Figure S6A). The CCK8 assays revealed a notable reduction in cell proliferation upon FAK knockdown. This effect was reversed by the reintroduction of FAK WT, but not FAK C456S (Figure S6B), as further confirmed by colony formation assays (Figure S6C). Moreover, the decreased ability of invasion and migration caused by FAK silencing was rescued by FAK WT, but not by FAK C456S (Figure S6D-G). These results demonstrate that ZDHHC5-mediated FAK S-palmitoylation promotes GBM cell proliferation and EMT.

### ZDHHC5 promotes GBM development in vivo

**Fig. 6:**
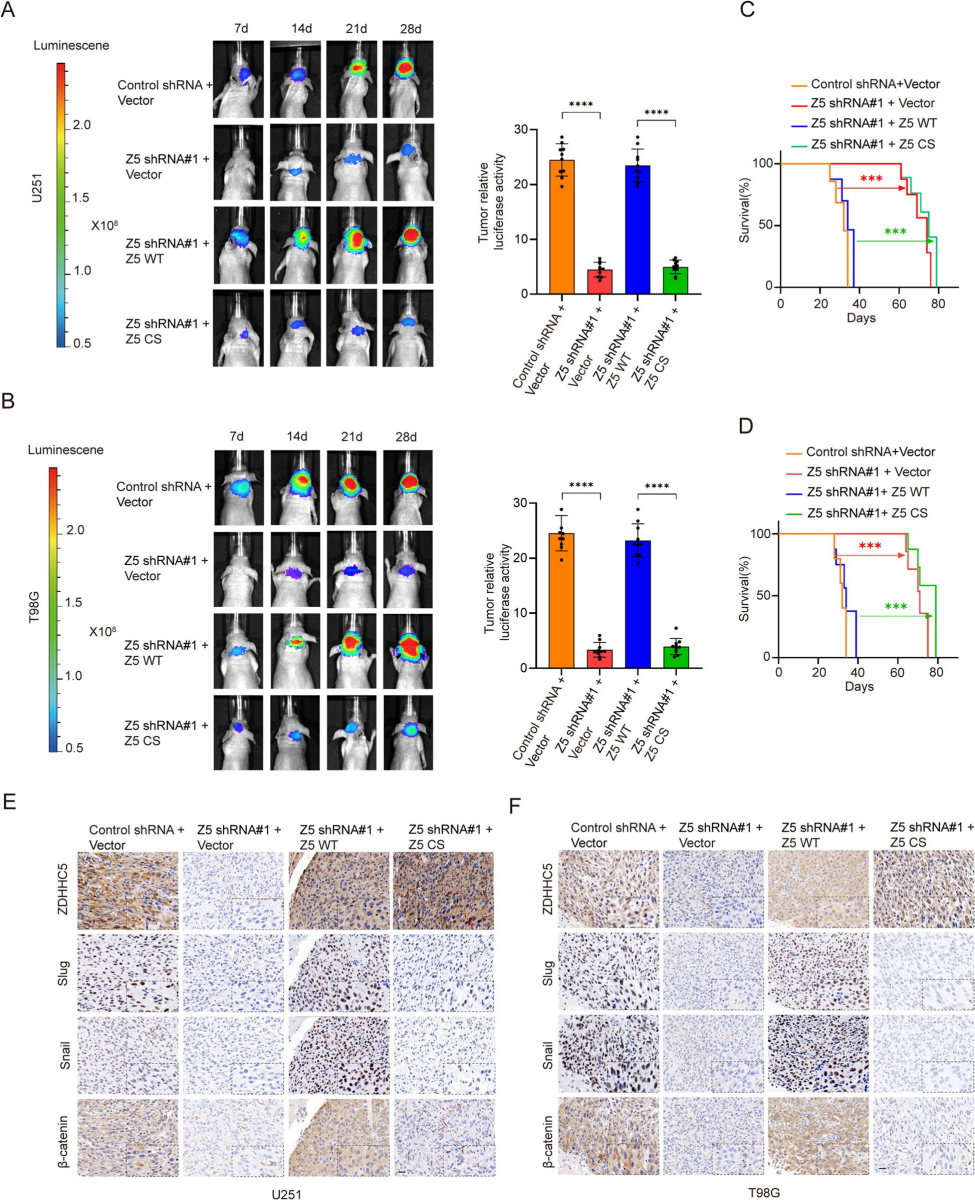
ZDHHC5 promotes GBM development in vivo. **A** and **B** Luciferase-expressing ZDHHC5-knockdown U251 and T98G cells were injected into athymic nude mice (*n* = 10). The mice were subjected to IVIS scanning on days 7,14,21,28 after the injection of tumor cells. Representative bioluminescent images of intracranial GBM xenografts are shown on the left. Quantification of bioluminescent images (day = 28) is shown on the right. **C** and **D** Kaplan‒Meier survival curves of mice intracranially injected with U251 and T98G cells with the indicated modifications (*n* = 10). *P* values were calculated using the two-tailed Student's t test for (**A**, **B**) and the log-rank (Mantel‒Cox) test for (**C**, **D**). **P < 0.01, ***P < 0.001, ****P < 0.0001. **E** and **F** IHC images of the indicated proteins are shown in consecutive brain sections. Scale bars, 20 µm

To investigate the role of ZDHHC5 in vivo, we intracranially implanted luciferase-expressing U251 and T98G cells with or without knockdown of endogenous ZDHHC5 and re-expression of the ZDHHC5 WT or ZDHHC5 C134S mutant counterpart into athymic nude mice. Bioluminescence imaging revealed that ZDHHC5 depletion significantly decreased tumor growth in mice following the injection of U251 and T98G cells (Fig. 6A and B). This intervention led to a considerable increase in the survival time (Fig. 6C and D). Moreover, the expression of EMT-associated proteins, such as Snail, Slug, and β-catenin was markedly downregulated upon ZDHHC5 knockdown, as demonstrated by the IHC assay (Fig. 6E and F). However, reconstitution of ZDHHC5 WT but not ZDHHC5 C134S prevented these alterations (Fig. 6A-F). Collectively, these results indicate that ZDHHC5 is required for GBM carcinogenesis.

### ZDHHC5 expression positively correlates with S-palmitoylated FAK in clinical glioma samples

**Fig. 7:**
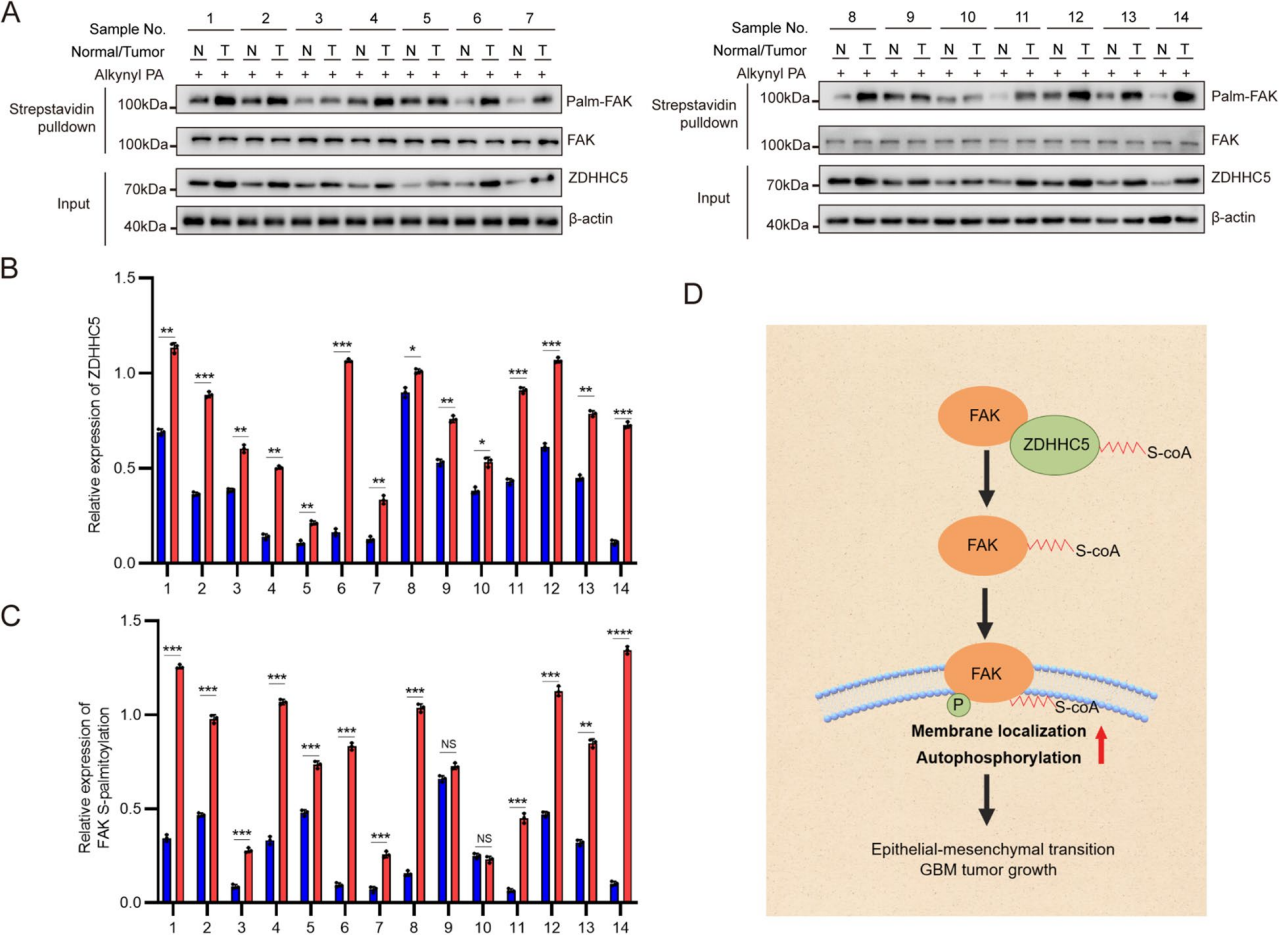
ZDHHC5 expression positively correlates with FAK expression in clinical glioma samples. **A** The S-palmitoylation of FAK in glioma samples (T) and their paired normal tissues (N) were analyzed by metabolic incorporation assay. **B** and **C** Relative expression levels of ZDHHC5 (**B**) and S-palmitoylation of FAK (**C**) from (**A**). Data are represented as the mean ± SD (*n* = 3). Statistical analysis was performed using Student’s t test, *p < 0.05; **p < 0.01; ***p < 0.001; ****p < 0.0001. **D** Schematic representation of the study model. ZDHHC5-catalyzed palmitoylation of FAK leads to its membrane localization and activation, subsequently promoting EMT and the development of GBM

To evaluate the clinical relevance of ZDHHC5 and S-palmitoylated FAK, we used metabolic incorporation assay to assessed the S-palmitoylation level of FAK in 14 paired tumors (Fig. 7A). Both the protein level of ZDHHC5 and S-palmitoylation level of FAK were markedly elevated in glioma tissue compared to normal tissue (Fig. 7B and C).

## Discussion

Previous research has demonstrated that FAK remains in an autoinhibited state within the cytosol and is activated upon interaction with the membrane [35]. This observation led to the hypothesis that proper localization of FAK to the membrane plays a critical role in facilitating its related biological processes. Here, we show that ZDHHC5 mediates the S-palmitoylation of FAK at Cys456 to maintain the membrane binding of FAK. Furthermore, the autophosphorylation of membrane-localized FAK and activation of downstream signaling pathways are increased, consequently promoting GBM development (Fig. 7D). These results emphasize the significance of protein subcellular localization during cancer progression [32, 42, 43].

Palmitoylation, which refers to the reversible covalent attachment of palmitate molecules to cysteine residues of proteins, is widely recognized as the most extensively studied protein lipidation process [44]. It plays a crucial role in regulating various physiological processes within the cell and influences the stability, conformation, localization, and interactions of proteins [32, 45–49]. The S-palmitoylation of FAK was previously discovered via proteomic analysis [50]. However, the impact of S-palmitoylation on the function of FAK remains unexplored. In this study, we determined that ZDHHC5-mediated S-palmitoylation of FAK is important for its biological function, especially in regulating its membrane localization and phosphorylation in human GBM cells. Importantly, in our study, the palmitoylation-deficient FAK mutant was not able to localize normally at the cell membran, which resulted in a decrease in its phosphorylation, highlighting the importance of *S*-palmitoylation in the FAK signalling pathway.

Significantly, our results revealed that knockdown of ZDHHC5 effectively suppressed the migration, invasion, and proliferation of GBM cells in vitro, and inhibited GBM development in vivo. These findings suggest that ZDHHC5 promotes the malignant development of GBM.

Accumulating evidence indicates that epithelial-mesenchymal transition, characterized by the loss of cell polarity, loss of cell–cell adhesion, and alterations in the expression of cell surface and cytoskeletal proteins, plays a crucial role in tumor invasion and metastasis [51, 52]. During EMT, the expression of epithelial markers, such as E-cadherin, is reduced, while the expression of mesenchymal markers, such as N-cadherin, Snail, Slug, and β-catenin, is increased [51]. However, it is not yet clear whether palmitoylation is associated with EMT. In this study, we found that GBM cell migration and invasion dramatically decreased via suppression of EMT following ZDHHC5 knockdown. Reconstitution with expression of ZDHHC5 WT but not ZDHHC5 C134S restored the expression of EMT-associated proteins, implying that ZDHHC5 enhances invasive and tumorigenic properties by facilitating EMT in GBM cells.

## Conclusions

In summary, our findings reveal the significance of ZDHHC5-mediated FAK S-palmitoylation as a critical mechanism in the development of GBM. Targeting the ZDHHC5/FAK axis may be a novel and potentially effective treatment approach for GBM.

### Supplementary Information

**Additional file 1: Supplementary Figure 1. **S-palmitoylation maintains membrane localization of FAK. **Supplementary Figure 2.** ZDHHC5 regulates FAK S-palmitoylation by directly binding to FAK. **Supplementary Figure 3.** ZDHHC5 maintains FAK membrane localization and activation via S-palmitoylation. **Supplementary Figure 4.** FAK is palmitoylated at Cys456. **Supplementary Figure 5.** ZDHHC5-mediated FAK S-palmitoylation promotes cell proliferation, cell invasion and EMT in vitro. **Supplementary Figure 6.** ZDHHC5-mediated FAK S-palmitoylation promotes cell proliferation, cell invasion and EMT in vitro.

**Additional file 2.**

## Data Availability

All data needed to evaluate the conclusions in the paper are present in the paper and/or the Supplementary Materials. Additional data related to this paper may be requested from the authors.

## Abbreviations

GBM: Glioblastoma
FAK: Focal adhesion kinase 1
PAT: Protein-acyl transferase
EMT: Epithelial-mesenchymal transition
HAM: Hydroxylamine
2-BP: 2-Bromopalmitate
APE: Acyl-PEG exchange
PalB: Palmostatin
