# Supplementary material for: ZDHHC5-mediated S-palmitoylation of FAK promotes its membrane localization and epithelial-mesenchymal transition in glioma
Source: Cell Commun Signal. 2024 Jan 17;22:46. doi: 10.1186/s12964-023-01366-z (PMC10795333; doi:10.1186/s12964-023-01366-z)
Supplement: Supplementary file 2 — Additional file 1: Supplementary Figure 1. S-palmitoylation maintains membrane localization of FAK. Supplementary Figure 2. ZDHHC5 regulates FAK S-palmitoylation by directly binding to FAK. Supplementary Figure 3. ZDHHC5 maintains FAK membrane localization and activation via S-palmitoylation. Supplementary Figure 4. FAK is palmitoylated at Cys456. Supplementary Figure 5. ZDHHC5-mediated FAK S-palmitoylation promotes cell proliferation, cell invasion and EMT in vitro. Supplementary Figure 6. ZDHHC5-mediated FAK S-palmitoylation promotes cell proliferation, cell invasion and EMT in vitro. [file 12964_2023_1366_MOESM1_ESM.pdf]

Supplementary Figure. 1

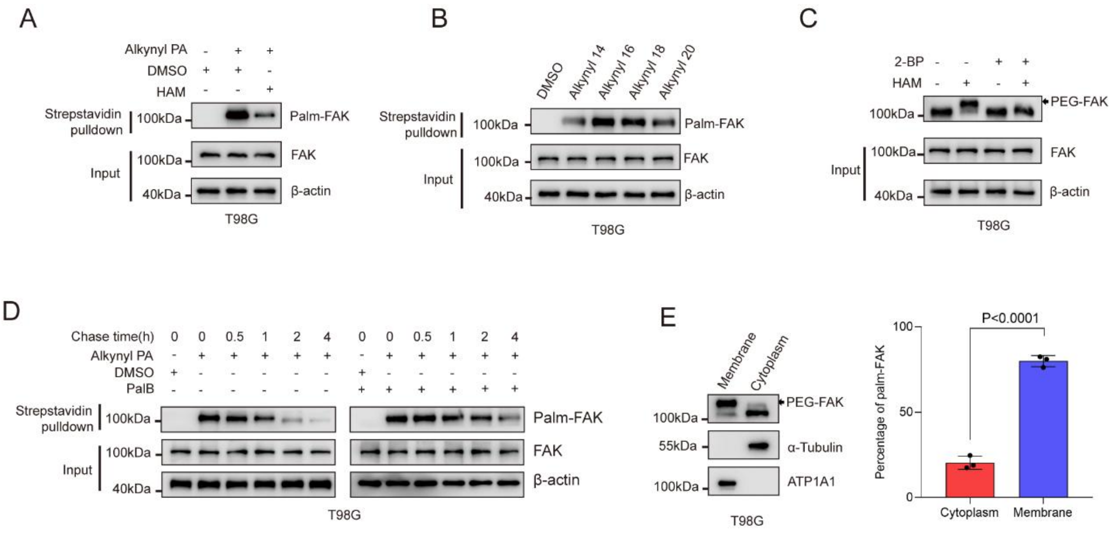

Supplementary Figure. 1 S-palmitoylation maintains membrane localization of FAK (A) FAK palmitoylation was examined in lysates obtained from T98G cells that were metabolically treated with a palmitoylation probe (50 μM alkynyl palmitic acid [PA]) for a duration of 4 hours. The analysis was carried out through click reaction and streptavidin bead pulldown, both in the absence and presence of hydroxylamine (HAM). The subsequent immunoblotting (IB) was performed using indicated antibodies. (B) FAK fatty acylation levels were investigated using different chemical reporters of fatty acylation, ranging from Alk-C14 to Alk-C20. (C) The APE assays were conducted to examine the levels of FAK palmitoylation in T98G cells following treatment with 50 μM of 2-BP, both in the absence and presence of HAM. (D) FAK palmitoylation studies were conducted in T98G cells that were metabolically labeled with 50 μM alkynyl PA for a duration of 4 hours. The cells were treated either in the absence or presence of PalB (5 μM). After the specified time period, the cells were collected for further analysis of FAK palmitoylation. The analysis involved the use of streptavidin bead pulldown to isolate acylated FAK, followed by IB. (E) FAK

palmitoylation was analyzed using the APE assay after fractionation to distinguish between the cytoplasmic and membrane fractions. ATP1A1 and α-tubulin were used as controls for the membranal and cytoplasmic fractions, respectively. Quantification of FAK palmitoylation percentage in the cytoplasm and membrane in APE assays. All the data are presented as the mean ± SD, n=three independent experiments, and two-tailed Student's t test.

Supplementary Figure. 2

A

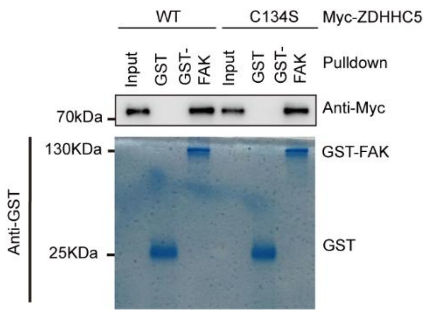

Supplementary Figure. 2 ZDHHC5 regulates FAK S-palmitoylation by directly binding to FAK (A)Myc-ZDHHC5 WT or ZDHHC5 C134S protein was purified and subsequently incubated with glutathione-Sepharose beads coupled with either GST or GST-FAK. The proteins that bound to the Sepharose beads were then analyzed by IB using the indicated antibodies. Recombinant GST-FAK protein was purified from bacteria, and its purity was confirmed by SDS–PAGE and Coomassie blue staining.

Supplementary Figure. 3

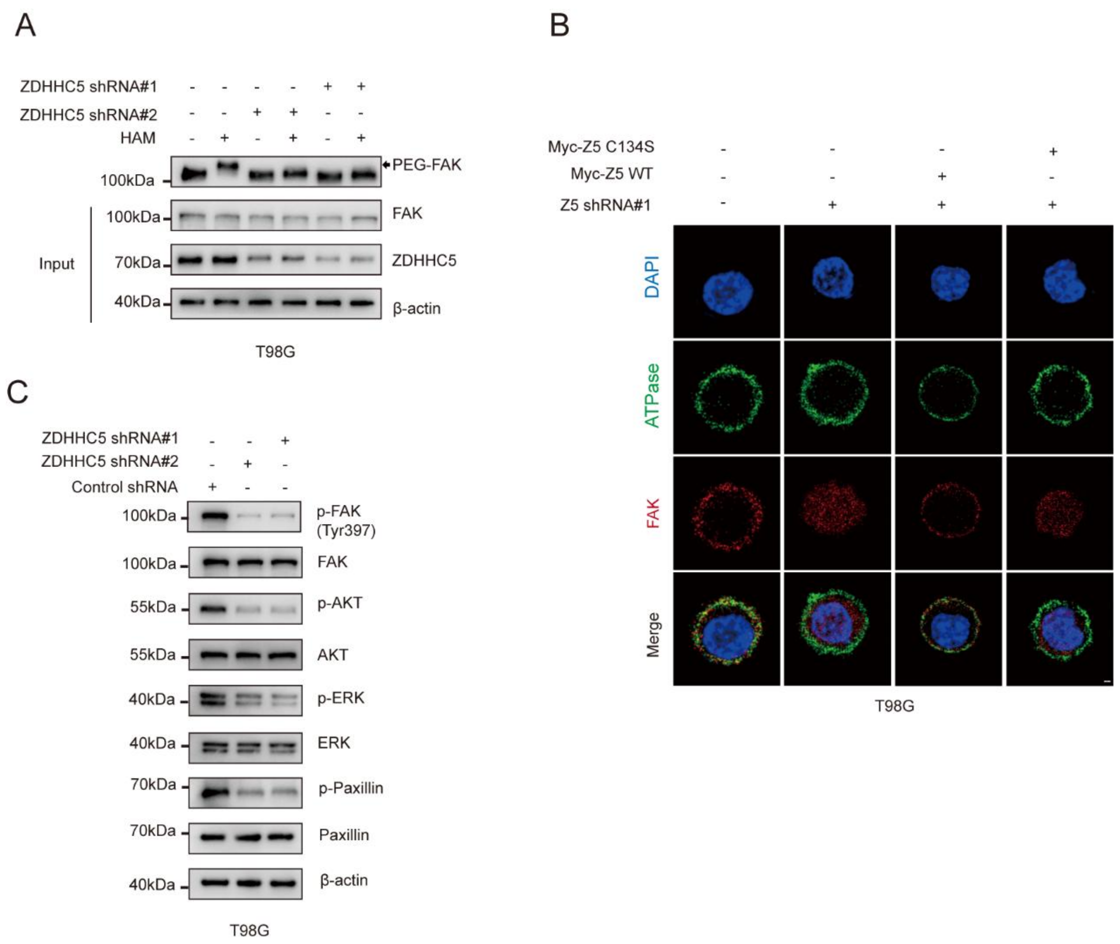

Supplementary Figure. 3 ZDHHC5 maintains FAK membrane localization and activation via S-palmitoylation (A) APE assay was performed to analyze the c-MET palmitoylation in ZDHHC5-knockdown T98G cells. (B) ZDHHC5-knockdown T98G cells were rescued with Flag-ZDHH5 WT or ZDHHC5 C134S, and endogenous FAK cellular localization was visualized by immunofluorescent staining using antibodies against FAK. Scale bar, 1 μm. (C) T98G cells were infected with lentiviruses expressing control shRNA or ZDHHC5 shRNAs, and then IB for indicated antibodies.

Supplementary Figure 4

A

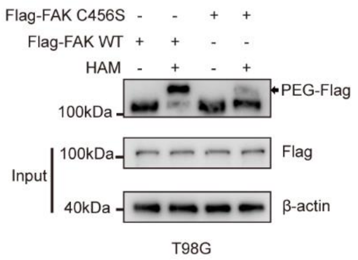

Supplementary Figure. 4 FAK is palmitoylated at Cys456 (A) APE assay was performed to analyze the FAK palmitoylation in T98G cells with indicated modifications.

Supplementary Figure. 5

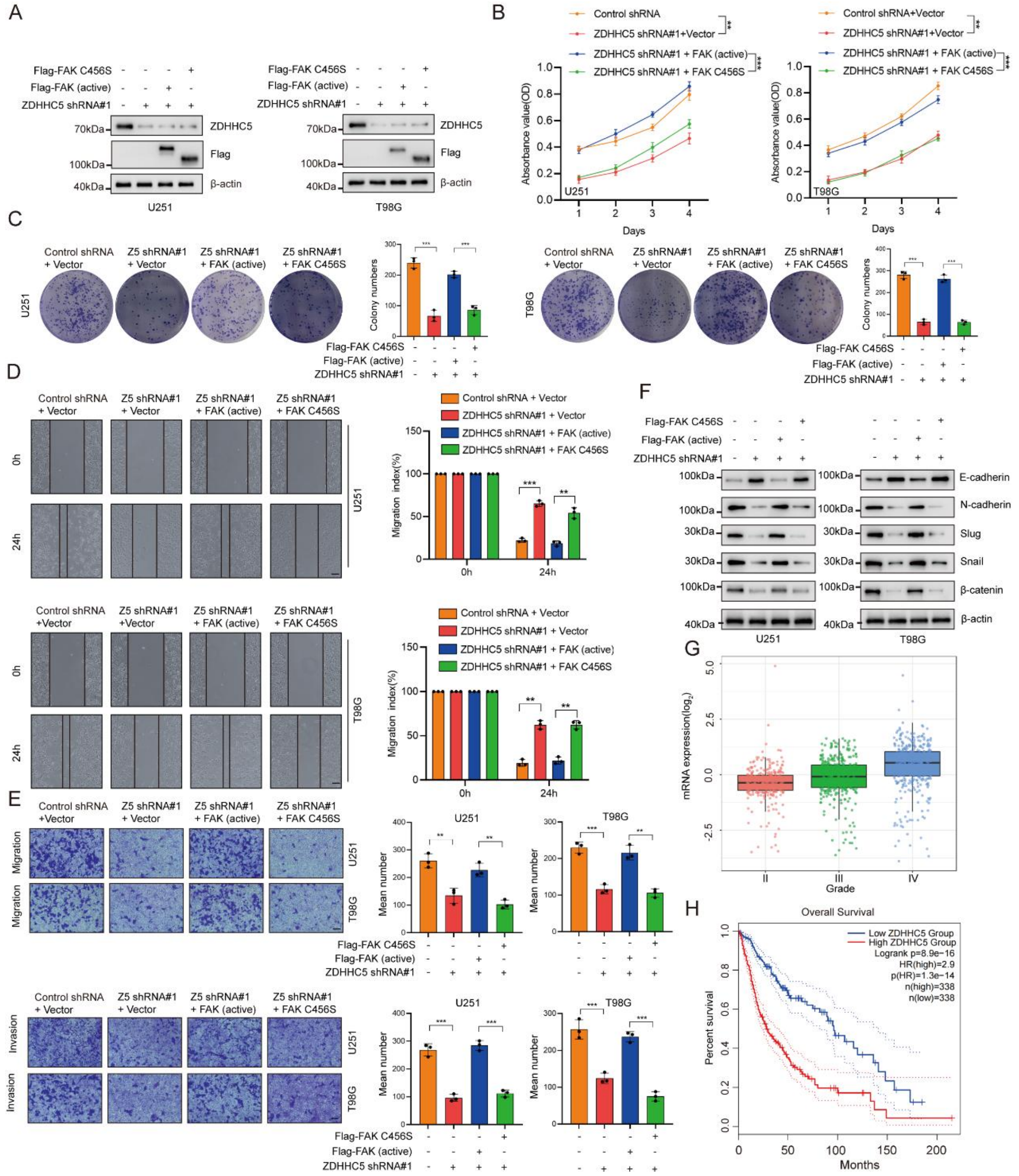

Supplementary Figure. 5 ZDHHC5-mediated FAK S-palmitoylation promotes cell proliferation, cell invasion and EMT in vitro. (A) IB for ZDHHC5 in U251 and T98G cells transfected with indicated modification. (B) CCK8 assays of U251 and T98G cells transfected with indicated modification. (C) Colony formation assay of U251 and T98G cells transfected with indicated modification. (D) Wound-healing assay of U251 and T98G cells transfected with indicated modification. scale bars: 25

μm. (E) Invasion of U251 and T98G cells transfected with indicated modification; scale bars: 100 μm. (F) IB for the indicated proteins in U251 and T98G cells transfected with indicated modification. Data are represented as the mean ± SD (n=3). Statistical analysis was performed using Student's t test, $^{**}p < 0.01$; $^{***}p < 0.001$; $^{****}p < 0.0001$. (G) Box plot analysis of ZDHHC5 mRNA levels in WHO II of glioma tissues (n = 291), WHO III of glioma tissues (n = 334), and WHO IV of glioma tissues (n = 388) from the CGGA database analyzed with the GlioVis website. (H) Kaplan-Meier analysis for ZDHHC5 expression in the TCGA datasets analyzed with the GEPIA 2 website.

Supplementary Figure. 6

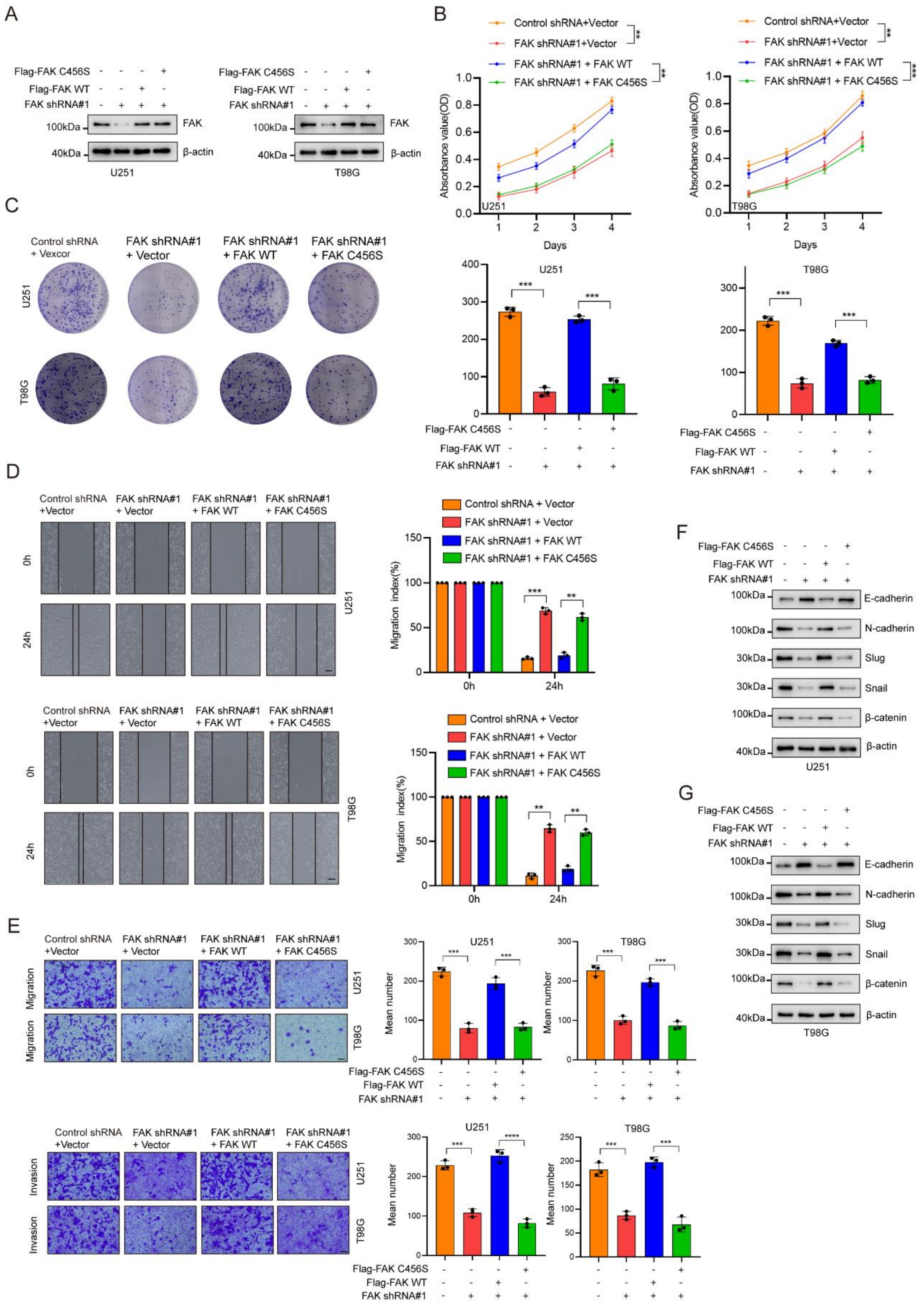

Supplementary Figure. 6 ZDHHC5-mediated FAK S-palmitoylation promotes cell proliferation, cell invasion and EMT in vitro. (A) IB for ZDHHC5 in U251 and T98G cells transfected with indicated modification. (B) CCK8 assays of U251 and

T98G cells transfected with indicated modification. (C) Colony formation assay of U251 and T98G cells transfected with indicated modification. (D) Wound-healing assay of U251 and T98G cells transfected with indicated modification. scale bars: 25 μm. (E) Invasion of U251 and T98G cells transfected with indicated modification; scale bars: 100 μm. (F) and (G) IB for the indicated proteins in U251 (F) and T98G (G) cells transfected with indicated modification. Data are represented as the mean ± SD (n=3). Statistical analysis was performed using Student's t test, $**p < 0.01$; $***p < 0.001$; $****p < 0.0001$.

Supplementary table 1

Gene name	siRNA target sequences
ZDHHC1	CAGCACGCACATGTCATTGAA
ZDHHC2	TAGCTACTGCTAGAAGTCTTA
ZDHHC3	AACATTGAGCGGAAACCAGAA
ZDHHC4	CAGGAGGGTCTCATTGACTCA
ZDHHC5	AGGGATTAGAGTGTGCTCCTA
ZDHHC6	AAGGCTAAAGATCGAATTCAG
ZDHHC7	CCCGTGGTTACTATGAATGTA
ZDHHC8	CGCGCCGTGTCTGATGTGTCA
ZDHHC9	CTCAACCAGACAACCAATGAA
ZDHHC11	CCATCAAGGTCCTGCCTGT
ZDHHC12	CAGATACTGCCTGGTGCTGCA

ZDHHC13	CAGCATAGTAGCCTTTCTATA
ZDHHC14	ACGCTTGTGGCCAGACTGCAA
ZDHHC15	GTGCTATGTGTGTGTTAAA
ZDHHC16	TAGCATCGAAAGGCACATCAA
ZDHHC17	TAGCGACATCTTATCCTATGA
ZDHHC18	AAGCCTGATGCCAGCATGGTA
ZDHHC19	CAGCAACTGGTATTTAACA
ZDHHC20	CAGCATTGACTTAGAGCTACA
ZDHHC21	AAGCGTAATTTGGACCTCTTT
ZDHHC22	CCCGCTGATAGCTGCGCAACA
ZDHHC23	AAGGATCAGGATTGCATGAAA
ZDHHC24	ACACCTAGACTCAGTAAGGAA
