# Supplementary material for: ZDHHC5-mediated S-palmitoylation of FAK promotes its membrane localization and epithelial-mesenchymal transition in glioma
Source: Cell Commun Signal. 2024 Jan 17;22:46. doi: 10.1186/s12964-023-01366-z (PMC10795333; doi:10.1186/s12964-023-01366-z)
Supplement: Supplementary file 3 — Additional file 2. [file 12964_2023_1366_MOESM2_ESM.pdf]

Fig3

A B C D E

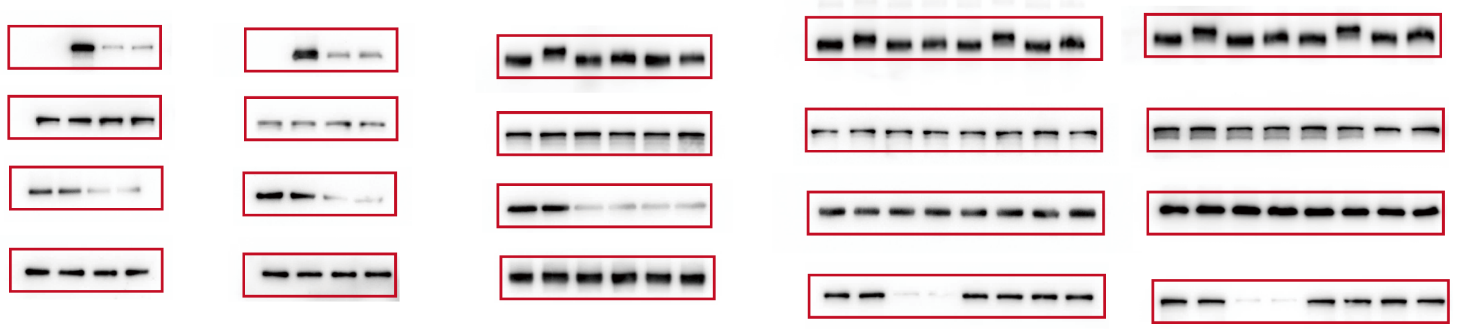

G H

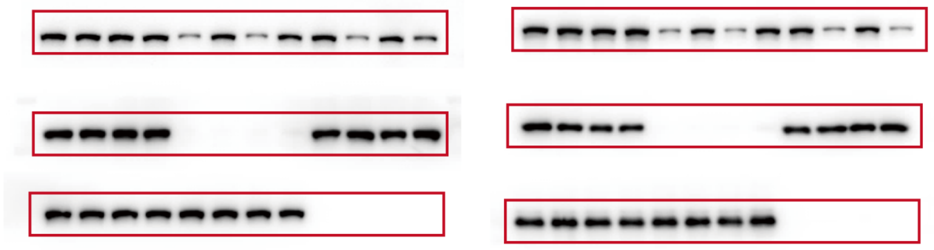

I

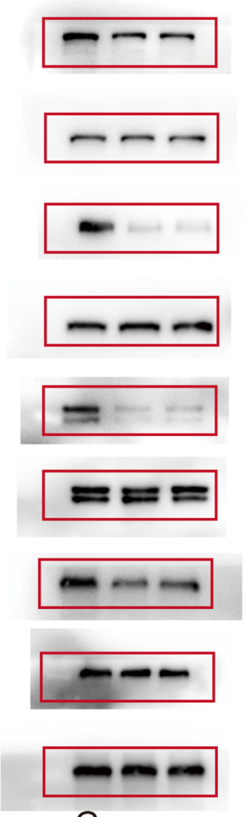

Fig4

A

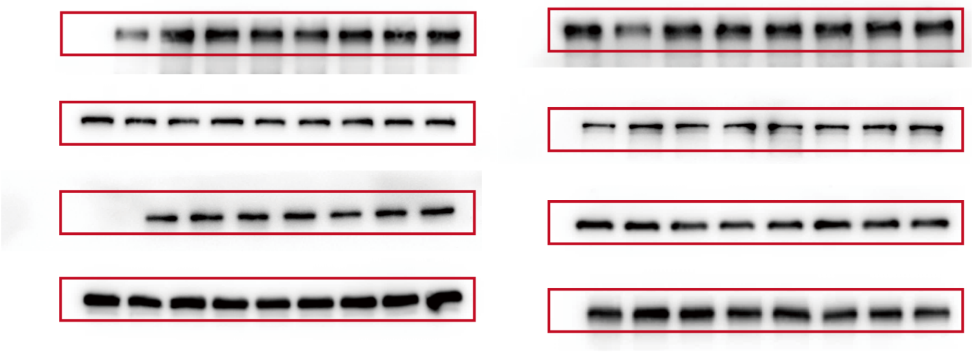

C D E F G

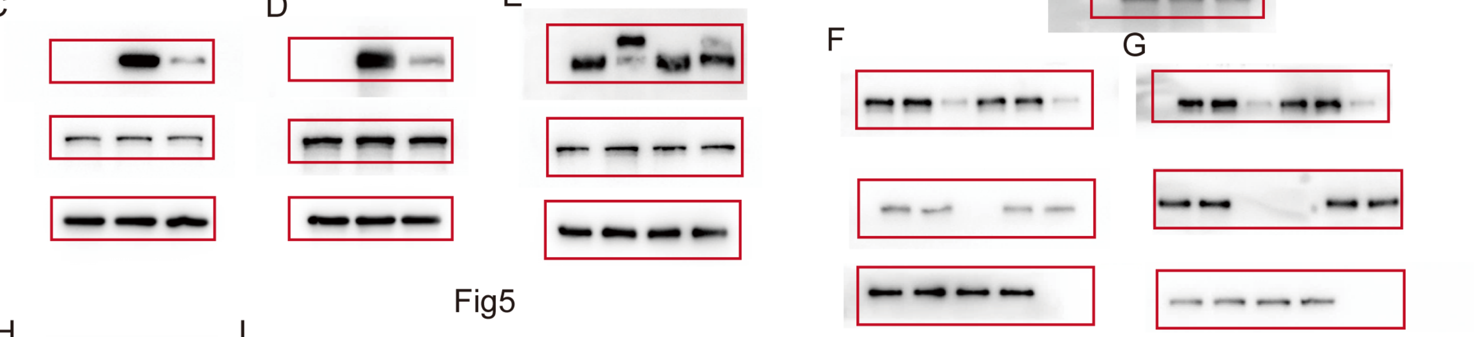

H I

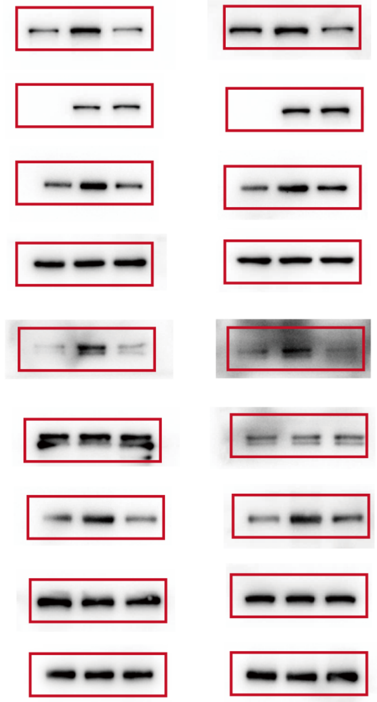

Fig5

A F

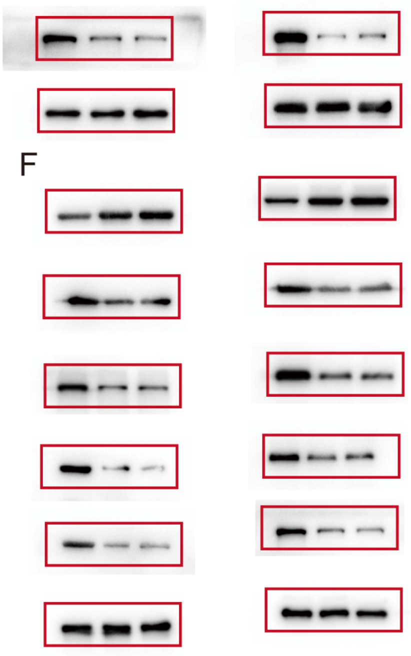

Fig7

A

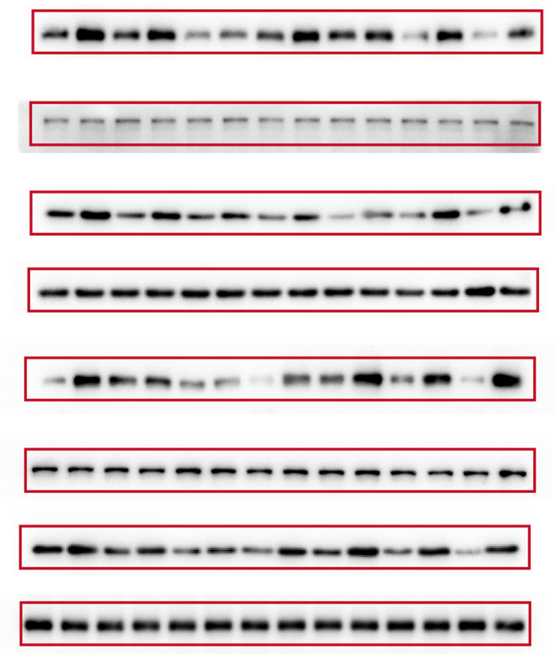

Figure S1

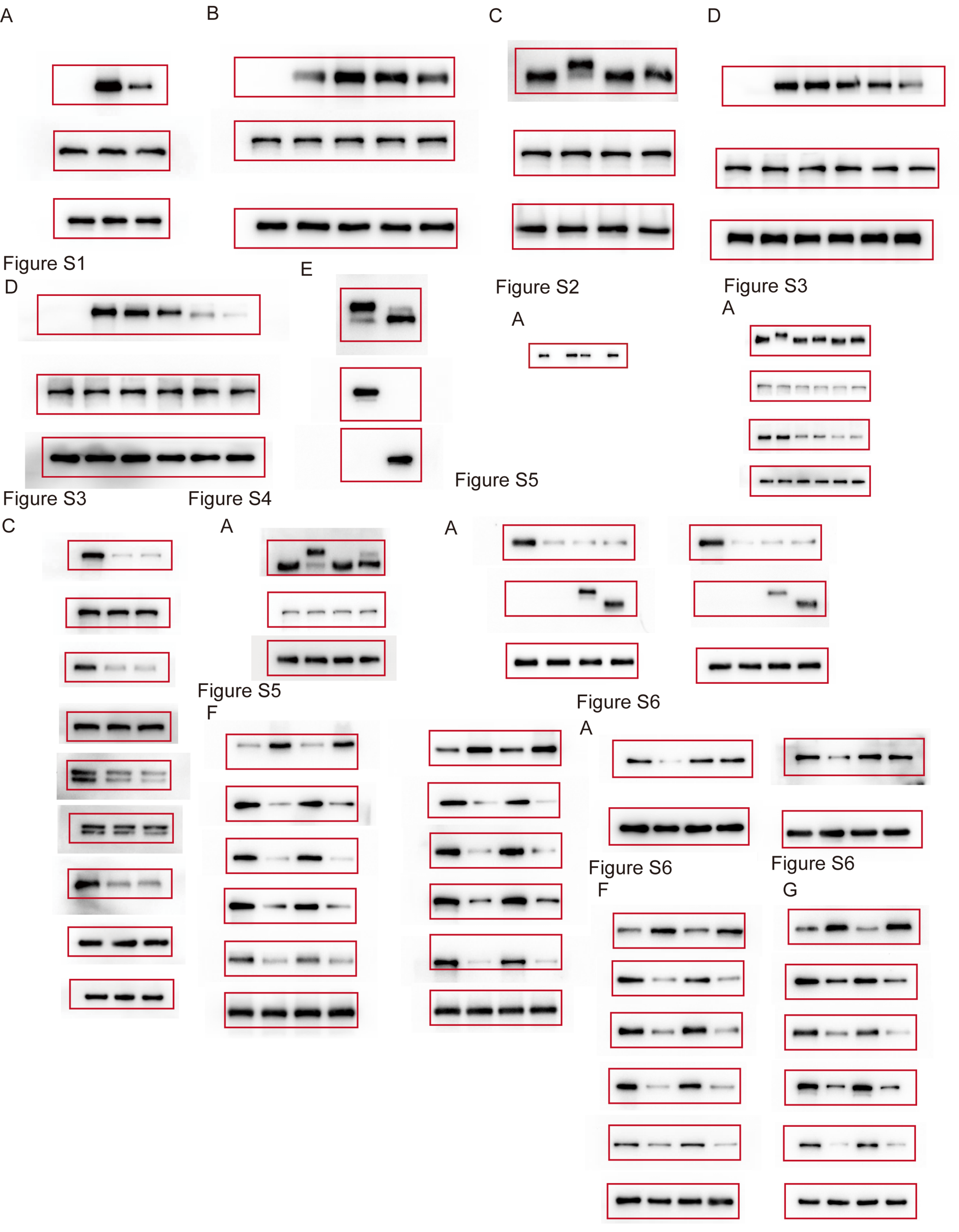
A
B
C
D
Figure S1
D
E
Figure S2
A
Figure S3
A
Figure S5
Figure S3
C
Figure S4
A
A
Figure S5
F
Figure S6
A
Figure S6
F
Figure S6
G

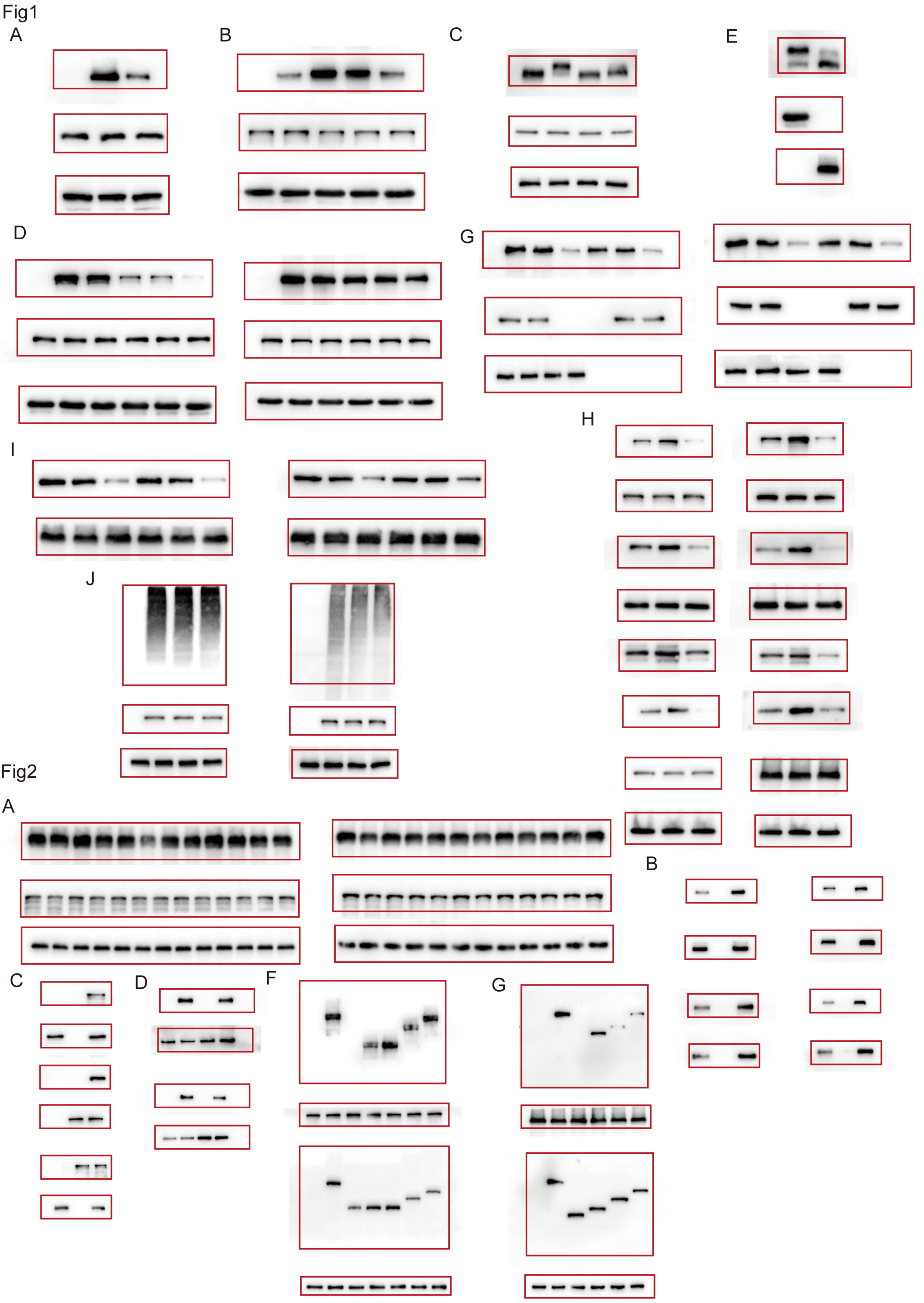
Fig1
A
B
C
E
D
G
H
I
J
Fig2
A
B
C
D
F
G
